# Supplementary figures and images for: Steady state evoked potential (SSEP) responses in the primary and secondary somatosensory cortices of anesthetized cats: Nonlinearity characterized by harmonic and intermodulation frequencies
Source: PLoS One. 2021 Mar 9;16(3):e0240147. doi: 10.1371/journal.pone.0240147 (PMC7943005; doi:10.1371/journal.pone.0240147)

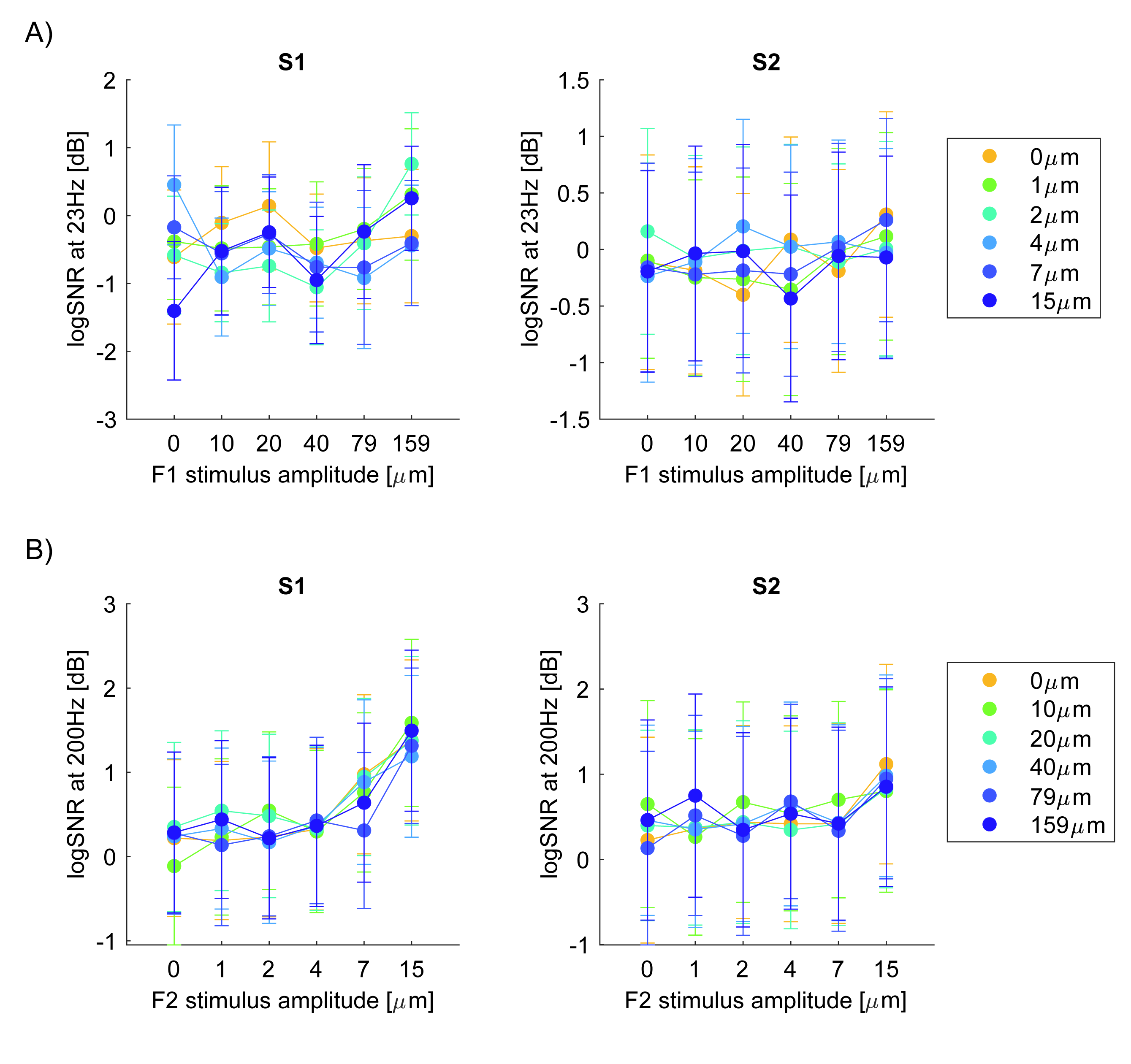

Supplement: S1 Fig — (A) logSNR at f1 = 23Hz as functions of F1 stimulus amplitudes for S1 and S2. We computed the mean and standard error across trials per bipolar channel in Session 1–1 and 1–2, which had 6 amplitude conditions for each F1 and F2 vibration stimulus. The point in the graph is the mean across the channels and the error bar is the mean of the standard errors across the channels. Colour encodes F2 stimulus amplitudes. (B) logSNR at f2 = 200Hz as functions of F2 stimulus amplitudes for S1 and S2. Colour encodes F1 stimulus amplitudes. (TIF) [file pone.0240147.s001.tif]

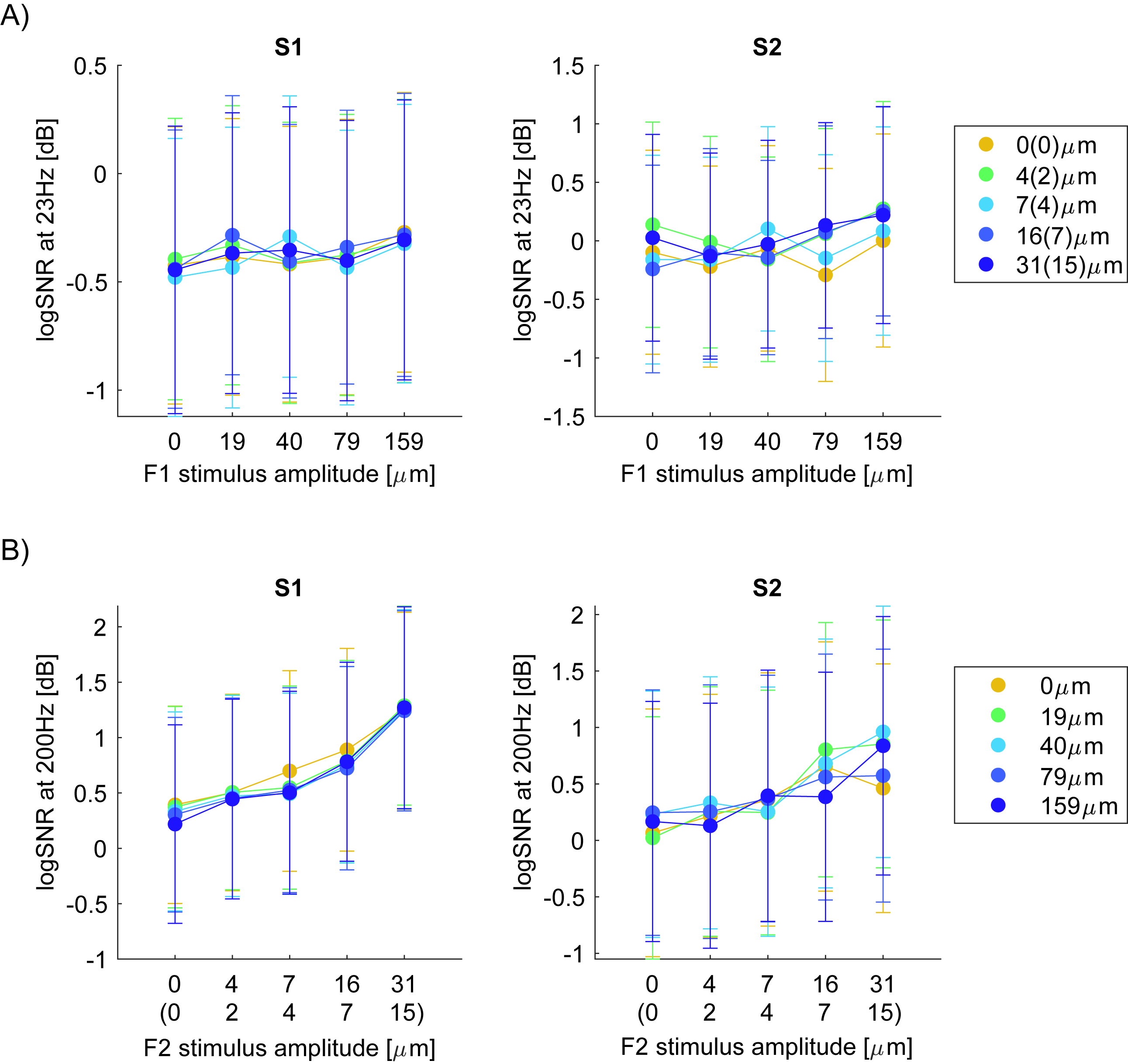

Supplement: S2 Fig — (A) logSNR at f1 = 23Hz as functions of F1 stimulus amplitudes for S1 and S2. The same format as S1 Fig. We included Session 1–3, 2–4, 2–5 and 2–6, which had 5 amplitude conditions. Note that Session 1–3 had different F2 stimulus amplitudes from the other sessions, these values that are shown in brackets. (B) logSNR at f2 = 200Hz as functions of F2 stimulus amplitudes for S1 and S2. (TIF) [file pone.0240147.s002.tif]

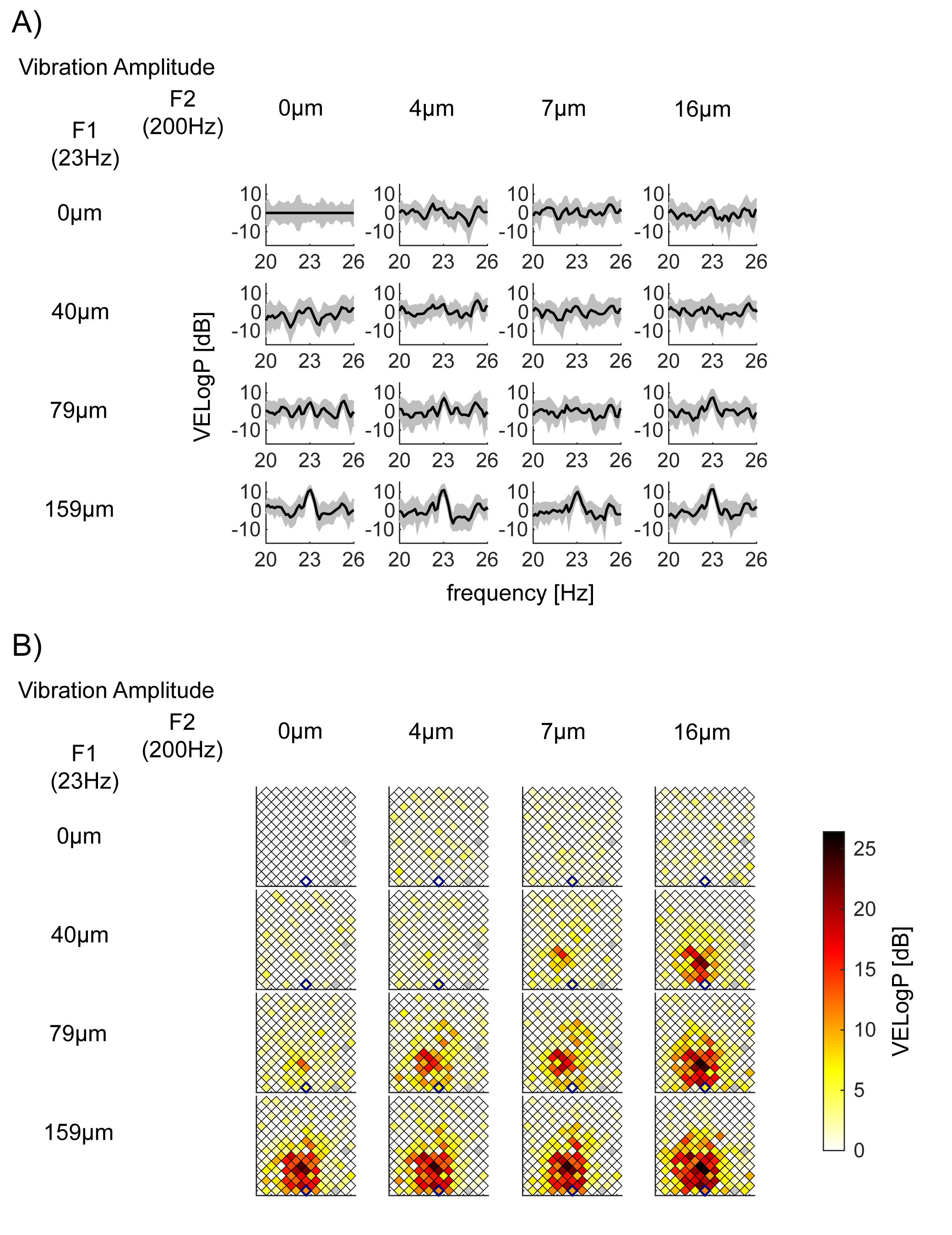

Supplement: S4 Fig — Shown with the same format as Fig 3. (A) VELogP of bipolar channel 176 in S1 (Session 2–2). This channel’s responses at f1 = 23Hz showed a significant main effect of F1 = 23Hz amplitude only. p-value (F1, F2, interaction) = (<10−5, 0.20, 0.042) with the corrected threshold 0.00016. (B) Spatial mapping of VELogP at f1 = 23Hz across all channels in S1 (Session 2–2). (TIF) [file pone.0240147.s004.tif]

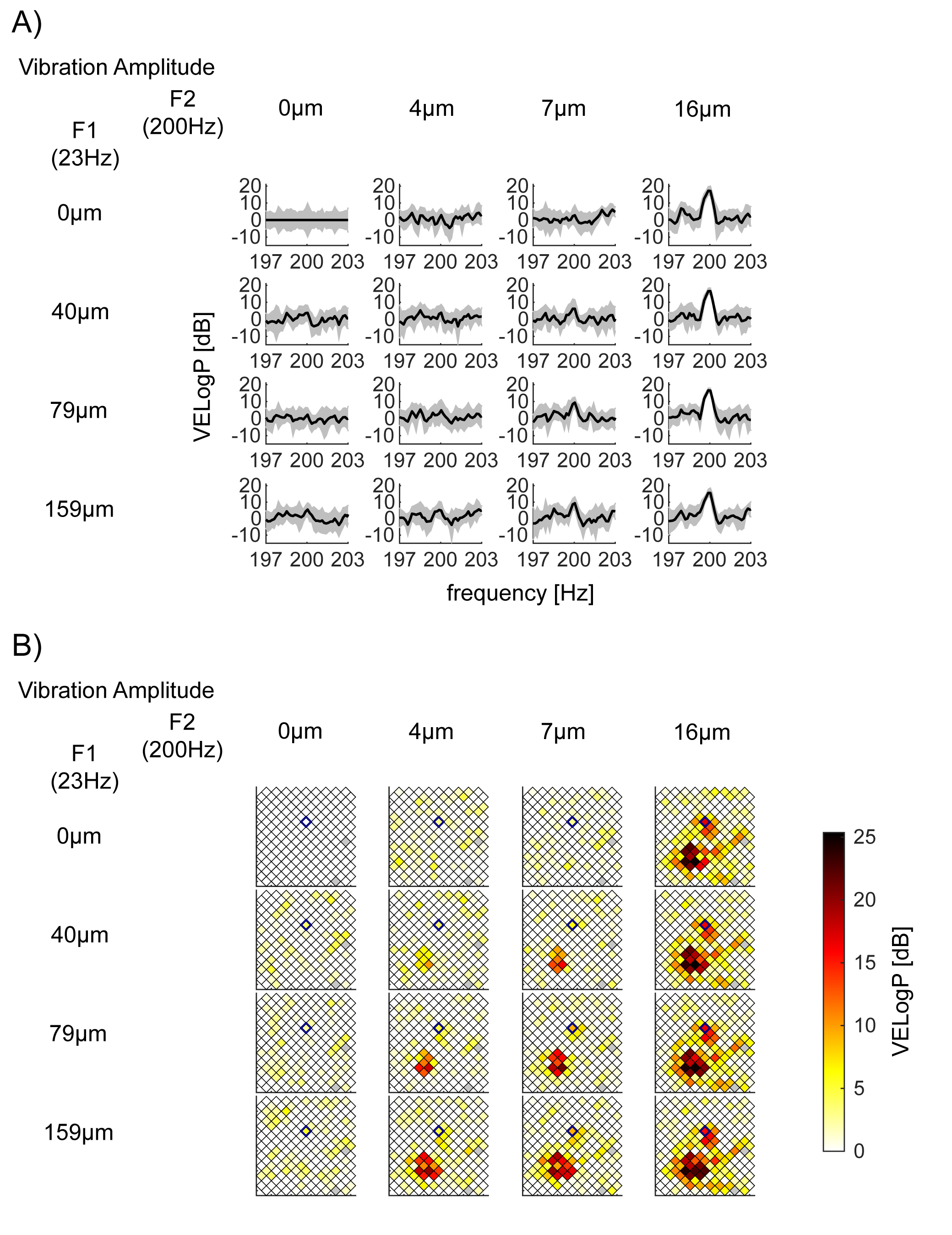

Supplement: S5 Fig — Shown with the same format as Fig 3. (A) VELogP of bipolar channel 122 in S1 (Session 2–2). This channel’s responses at f2 = 200Hz showed a significant main effect of F2 = 200Hz amplitude only. p-value (F1, F2, interaction) = (0.0033, <10−5, 0.045) with the corrected threshold 0.00016. (B) Spatial mapping of VELogP at f2 = 200Hz across all channels in S1 (Session 2–2). (TIF) [file pone.0240147.s005.tif]

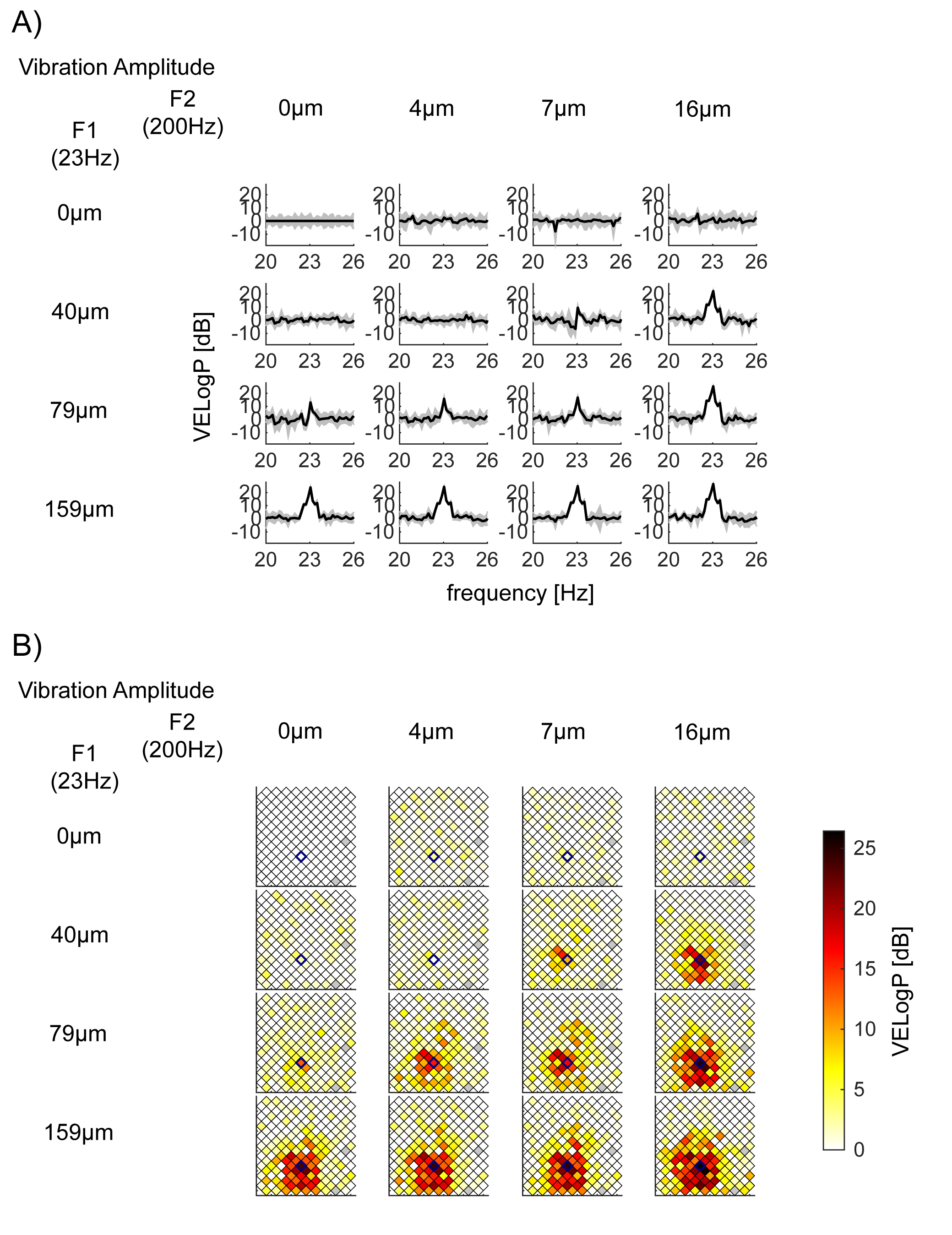

Supplement: S6 Fig — Shown with the same format as Fig 3. (A) VELogP of channel 43 in S1 (Session 2–2). This channel’s responses at f1 = 23Hz showed a significant main effect of F1 = 23Hz amplitude, the main effect of F2 = 200Hz, and their interaction. p-values (F1, F2, interaction) are all p<10−5 with the corrected threshold 0.00016. (B) Spatial mapping of VELogP at f1 = 23Hz across all channels in S1 (Session 2–2). (TIF) [file pone.0240147.s006.tif]

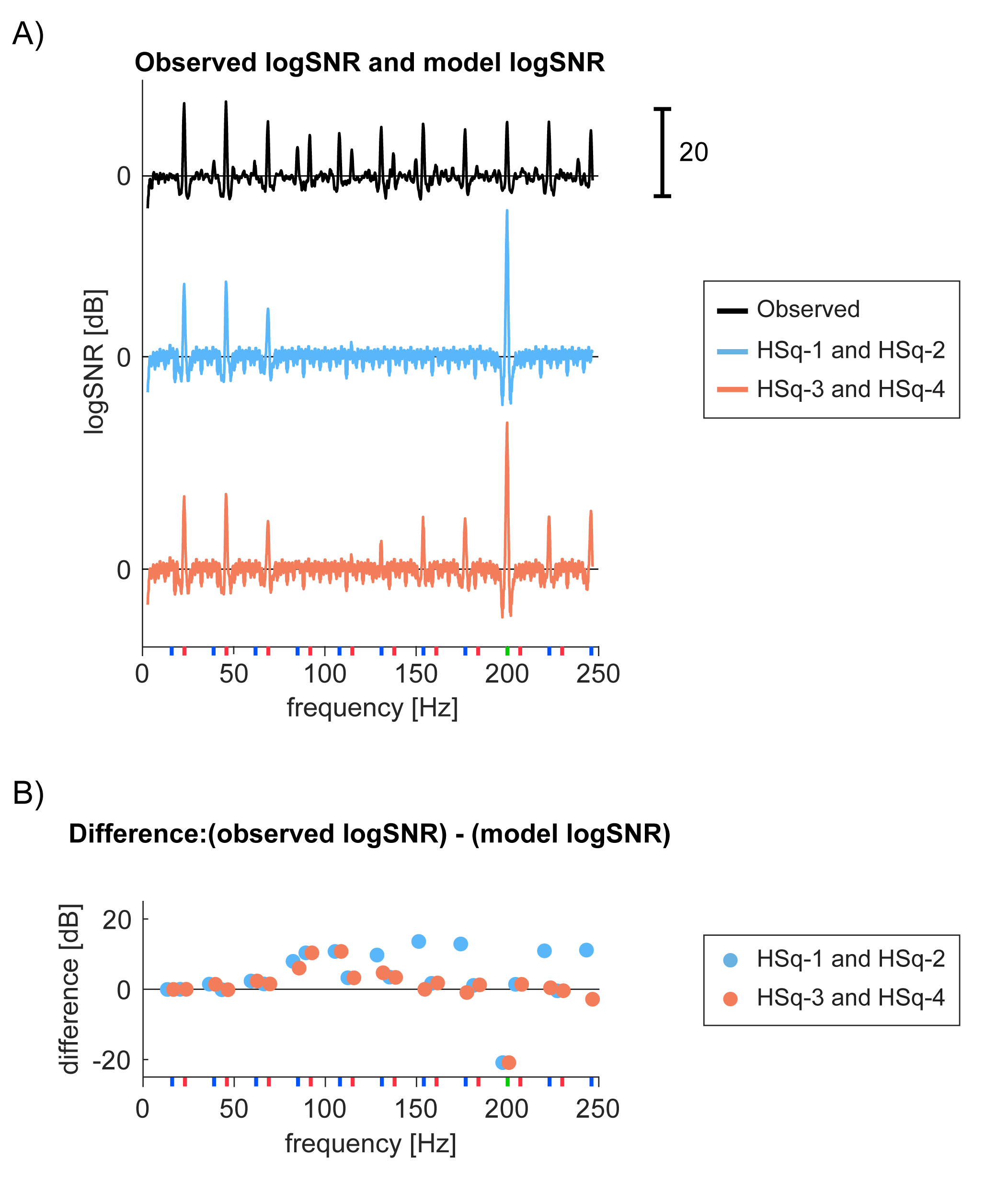

Supplement: S7 Fig — (A) Bipolar channel 43 in S1 (Session 2–2)’s logSNR (black) is compared to the optimally fitted models from four half squaring models. Adding HSq(XY) to two models (HSq-1 and HSq-3 in Fig 12) did not change the results and we showed four models in two colors: red for HSq-1 & HSq-2 and blue for HSq-3 & HSq-4. The best parameters for each model are the following. HSq-1 (red): -1.0HSq(X)+64HSq(Y), HSq-2 (red): -0.54HSq(X)+34HSq(Y) -1.4*10308HSq(XY), HSq-3 (blue): -0.94HSq(X)+38HSq(Y)+1.4HSq(X)HSq(Y) and HSq-4 (blue): -0.85HSq(X)+35HSq(Y)-1.4*10308HSq(XY)+1.3HSq(X)HSq(Y). The respective sums of logSNR differences from the observed logSNR across the frequencies of interest are 125 and 74. (B) Difference between the observed and the best model at the frequencies of interest. Color scheme is the same as in (A). (TIF) [file pone.0240147.s007.tif]

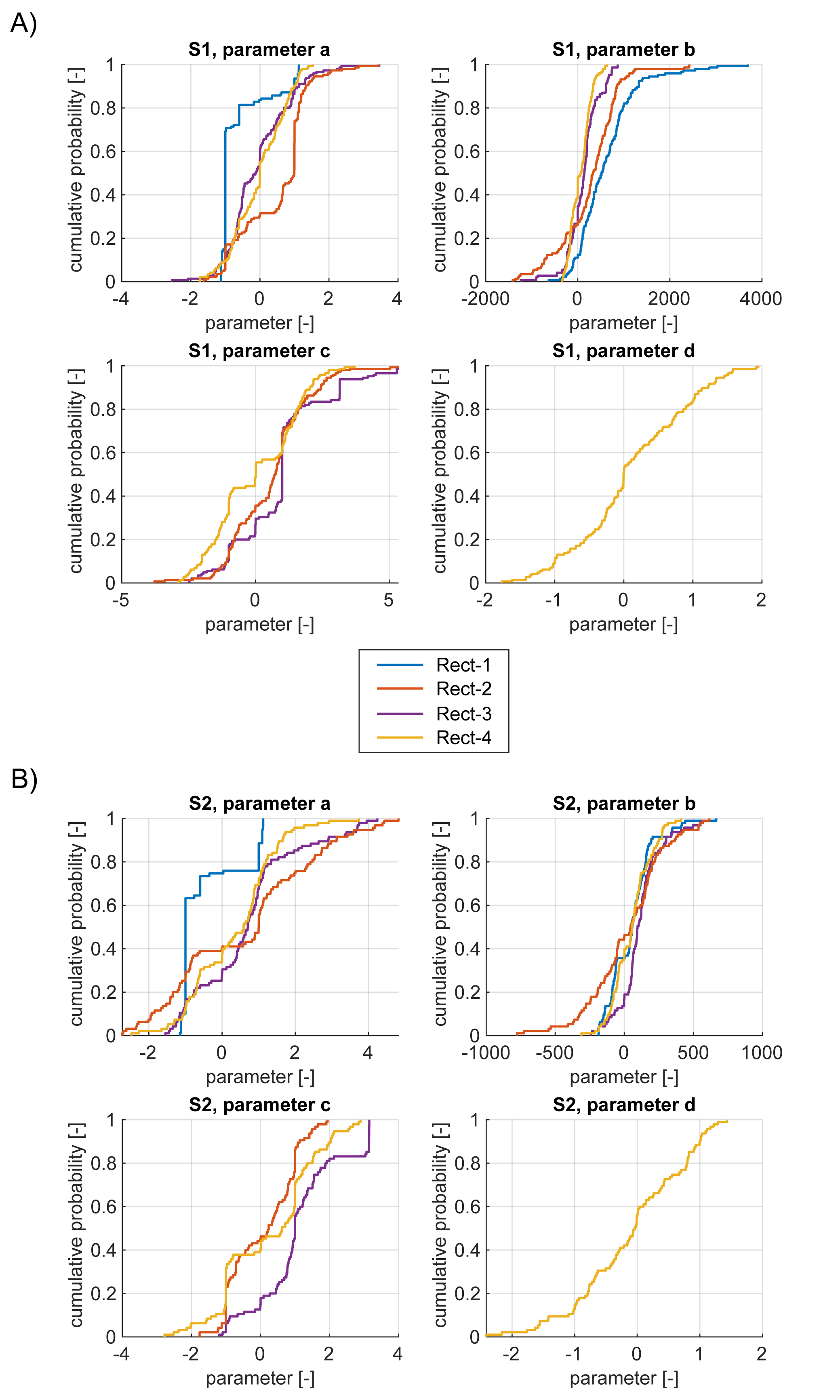

Supplement: S8 Fig — Cumulative probability distribution of each coefficient in S1 (A) and S2 (B). For a display purpose, we removed the top 3% and bottom 3% channels. This summarizes actual values of each parameter in rectification models. It also shows how adding a nonlinear component had an effect on other parameters. e.g. the top left panel in A shows that the model with Rect(X) (purple line) and the full model (yellow line) seemed to have similar the parameter a (i.e. coefficient of Rect(X) overall. On the other hand, the other two models (blue and red) seemed to have different parameters overall. (TIF) [file pone.0240147.s008.tif]

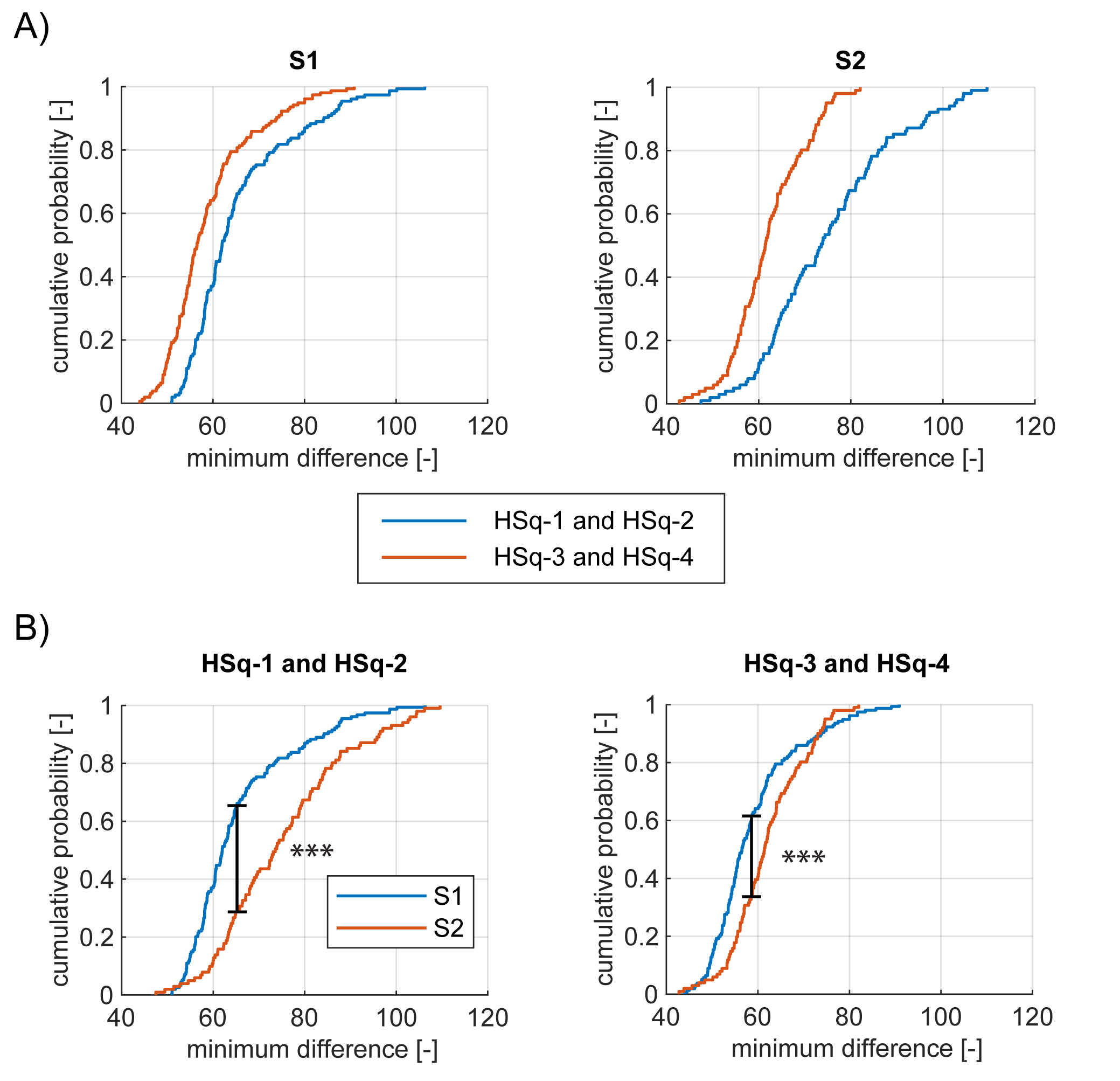

Supplement: S9 Fig — (A) Comparison of performance across four half squaring models based on the cumulative probability distributions of the minimum difference for S1 and S2 separately. Adding HSq(XY) did not change the results and we showed four models in two colors: red for HSq-1 and HSq-2 and blue for HSq-3 and HSq-4. (B) Comparison of each model’s performance between S1 and S2 based on the cumulative probability distributions of the minimum difference. We showed the two models giving the same distribution curves as one subplot. *** indicates p<0.001 according to Kolmogorov-Smirnov tests. (TIF) [file pone.0240147.s009.tif]

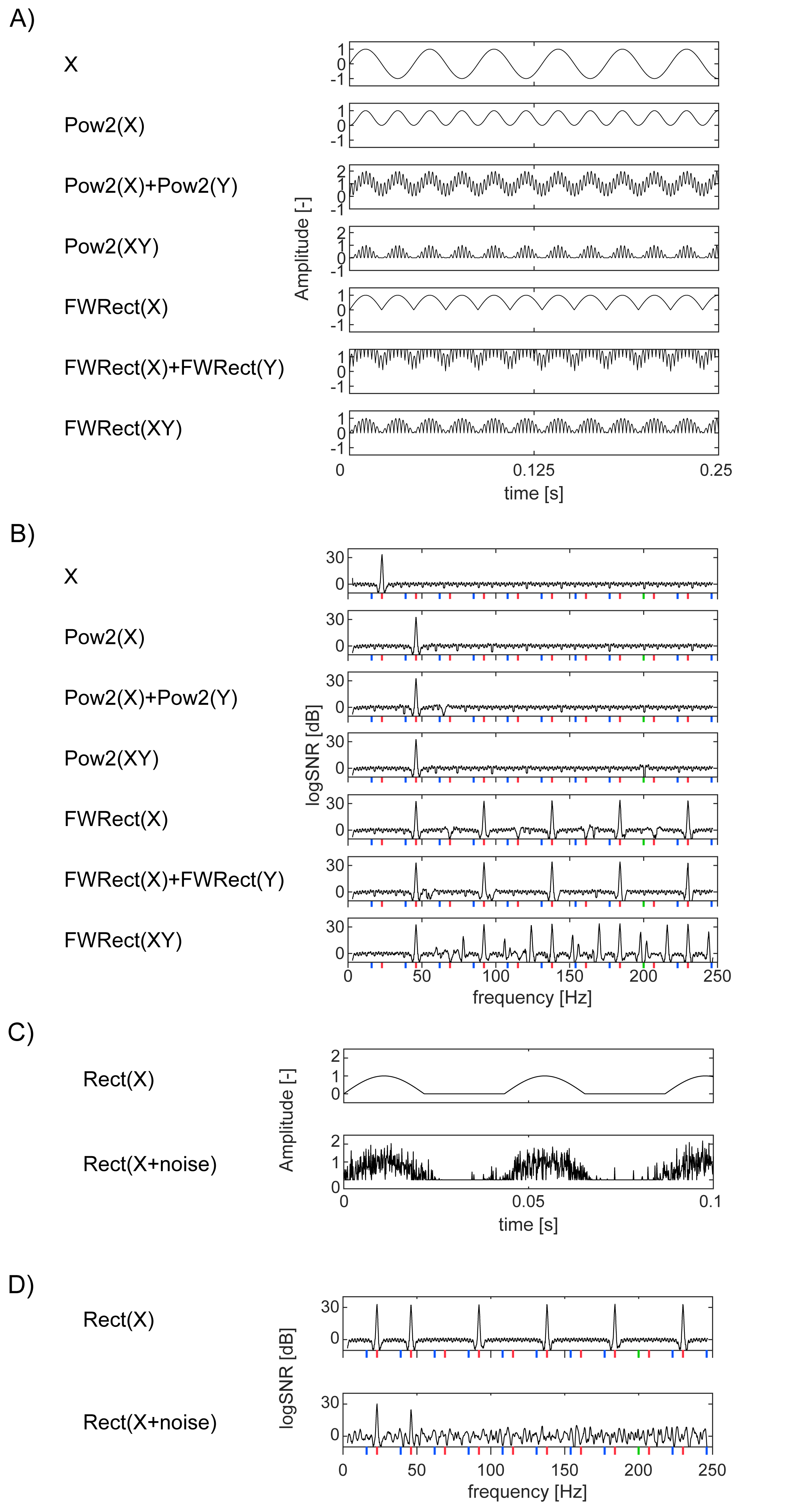

Supplement: S10 Fig — (A) Waveforms in the time domain (0 to 0.25s). X and Y are sinusoidal inputs at 23Hz and 200Hz, respectively. Pow2 represents the squaring (i.e. X2) and FWRect represents the full-wave rectification. Note that, for these operations, multiplication inside and outside of functions are identical with each other i.e. f(X)*f(Y) = f(X*Y). (B) Spectra of each waveform in the frequency domain. All of these models do not generate responses at fundamental frequencies. (C) Noise can suppress responses at harmonic frequencies in Rect(X). Waveforms in the time domain (0 to 0.1s). Noise is given by normal distribution N(0, 0.52) (as in the simulation reported in Gordon et al. 2019). (D) Spectra of each waveform in the frequency domain. Adding noise to the input suppresses responses at some harmonic frequencies. (TIF) [file pone.0240147.s010.tif]

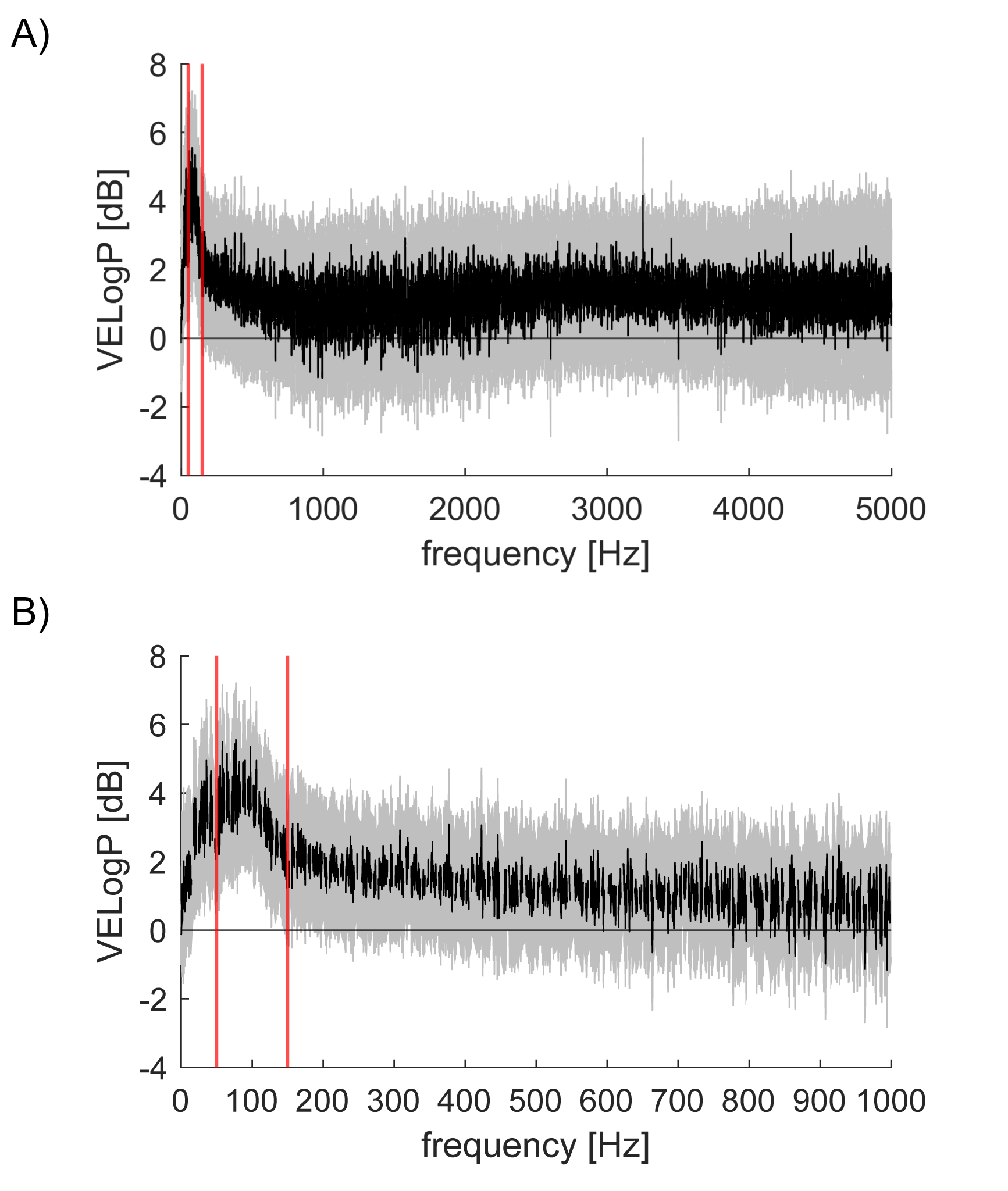

Supplement: S11 Fig — VELogP from top 10% channels in S2 across A) 0-5000Hz and B) 0-1000Hz). The top 10% channels in S2 showing higher HGP responses. We took the mean and standard error across trials for each channel and then took their respective means across channels. Red vertical lines show 50Hz and 150Hz. For a display purpose, we removed responses at fundamentals, harmonics and some intermodulation frequencies (TIF) [file pone.0240147.s011.tif]
