# Supplementary material for: Steady state evoked potential (SSEP) responses in the primary and secondary somatosensory cortices of anesthetized cats: Nonlinearity characterized by harmonic and intermodulation frequencies
Source: PLoS One. 2021 Mar 9;16(3):e0240147. doi: 10.1371/journal.pone.0240147 (PMC7943005; doi:10.1371/journal.pone.0240147)
Supplement: S1 Appendix — (PDF) [file pone.0240147.s012.pdf]

S1 Appendix. Line search optimisation technique.

For each model architecture, we used line search to find out a set of model parameters that minimized the difference between the model log*SNR* and the observed log*SNR*. (For the implementation of the following algorithm in MATLAB, see [1]).

We denote the difference that we tried to minimise as *d(i, p)* and define it as follows:

$$d(i,p)=\sum_{f\in F}\left|\log SNR_{model}^{p}(f)-\underline{\log SNR}_{record}^{i,[g_{\max},h_{\max}]}(f)\right| \quad (1)$$

d(i, p) is a function of channel *i* and a parameter *p* and it quantifies the sum of absolute differences of log*SNR* across a set of frequencies of our interest, *F. F* consists of fundamental (*f1* and *f2*), *f1* harmonic (46, 69, … up to 230), and intermodulation frequencies (16, 39, 62, 85, 108, 131, 154, 177, 223, 246). The underlined log*SNR* on the second term indicates the mean across trials for the stimulation condition with the maximum amplitude (i.e., g_{max} and h_{max}).

For line search, we used golden-section search [2]. This algorithm evaluates the objective function (i.e. *d(i, p)*) for four candidate parameters *p1, p2, p3,* and *p4*, where *p1, p2, p3, p4*$\in$*R* and *p1<p2<p3<p4*. If either *p2* or *p3* minimizes *d(i, p)*, the algorithm narrows down the search space by taking a new candidate within the search space. Otherwise, the algorithm expands the search space by taking a new candidate outside the search space. The golden-section search updates a candidate set of parameters (*p1, p2, p3, p4*) while keeping the same proportion, *r* : (1 - *r*), where *r* = *Φ* - 1 and *Φ* is the golden ratio.

Fig A1A demonstrates how the algorithm works. Here, in the first candidate set, *p2* minimizes *d(i, p)* which we denote as p_{min_1} = *p2*. The algorithm then set a new candidate parameter within *p1* and *p2* while keeping the golden ratio. The new candidate, *p5*, and the existing parameters (i.e. *p1, p2, p3*) constitute a new candidate set at the second step (the second row in Fig A1A, where p_{min_2} = *p3*). When the outermost value *p4* is selected at the *j*-th step, (e.g., p_{min_j} = *p4* at the third row in Fig A1A), a new candidate parameter will be set at greater than *p4* while keeping the golden ratio.

The algorithm terminates when the improvement of the *d(i, p)* slows down from (*j*-1)-th step to the *j*-th step. Specifically, we terminate the algorithm when $d(i, p_{min\_(j-1)}) - d(i, p_{min\_j}) < ET$, where *ET* means error tolerance value, which we explain below. To avoid spurious termination, we also imposed another condition that (*p4* - *p1)* < 0.0001, which ensures that our algorithm finds the minimum within a confined parameter range. If these criteria are satisfied at the *j*-th step, the algorithm returns the parameter giving the minimum *d(i, p)* in the *j*-th candidate set i.e. p_{min_j}.

When we considered a model architecture that had more than one coefficients, we sequentially optimized the coefficients in a fixed order. For example, for a model architecture with *aRect(X)+bRect(Y)*, we may first optimise coefficient *a* to be *a'* with the golden-section search. Then, we optimize *b* to be *b'* while keeping *a=a'*. We regard this sequential optimization of all coefficients as a single iteration of optimization (the orange lines and dots in Fig A1B), and we

iterated it 15 times. In the first iteration, we set initial candidate parameters as (*p1, p2, p3, p4*) = (-2, -2*r*, -2+2*r*, 0), (-1, 1-2*r*, -1+2*r*, 1), or (0, 2-2*r*, 2*r*, 2). In the *s*-th iteration when the previously optimised coefficient p_{s-1} from the (*s*-1)-th iteration is available, we started with (p_{s-1}, p_{s-1}+(1-*r*), p_{s-1}+*r*, p_{s-1}+1). While optimizing a parameter at the *s*-th iteration, we used the same error tolerance value *ET*(*s*). We set *ET*(*s*) to be 1 (for *s*=1….5), 0.1 (for *s*=6…10), and 0.01 (for *s*=11….15).

For any given channel *i* and a model architecture, we tried all possible combinations of initial parameter sets (3 possibilities) x all assignments of initial parameters (*N* possibilities for *N* parameters) x all orders of optimization (*N!* possibilities for *N* parameters) and reported the best across the combinations.

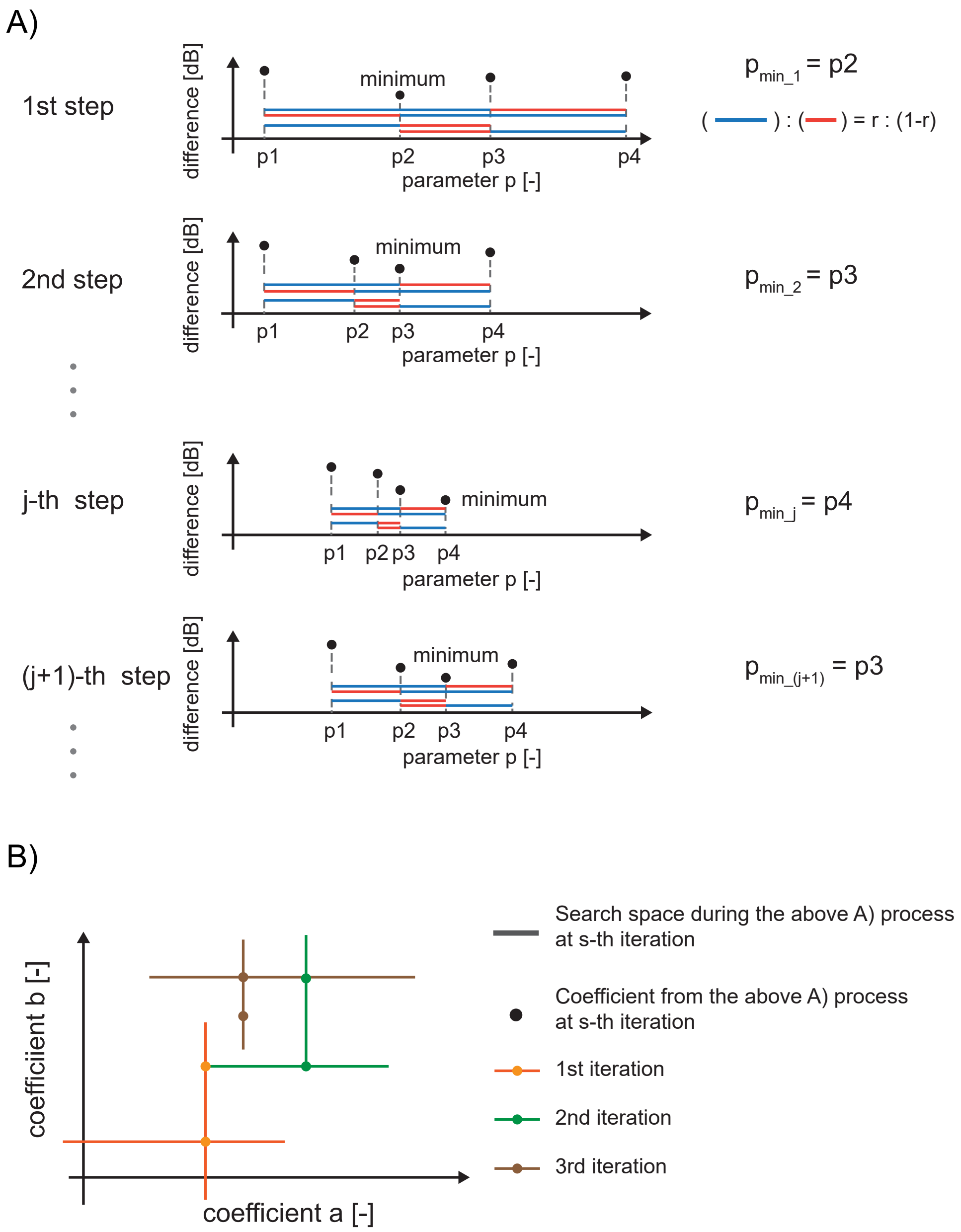

Fig A1. Explanation of Golden-section search algorithm.
(A) Single parameter optimization with Golden-section search. The algorithm takes four candidate points and computes their difference at each step. The point giving the minimum difference at the j-th step is shown as p_{min_j}. (B) Sequential optimization of two parameters. A line shows search space for each of single parameter optimization and a dot represents the optimized coefficient after the single parameter optimization depicted in A. This example optimised coefficient *a* first, then *b* at each iteration. Although we show up to the 3rd iteration here, we used 15 iterations to get results.

References

1. Kawshima Y, Li R. GitHub repository: yotaKawashima/mirror-tlab-unsw-catsomato. [cited 15 September 2020]. Available from: https://github.com/yotaKawashima/mirror-tlab-unsw-catsomato.git.
2. Scales LE. Introduction to non-linear optimization. New York: New York : Springer-Verlag; 1985.
