# Supplementary material for: Steady state evoked potential (SSEP) responses in the primary and secondary somatosensory cortices of anesthetized cats: Nonlinearity characterized by harmonic and intermodulation frequencies
Source: PLoS One. 2021 Mar 9;16(3):e0240147. doi: 10.1371/journal.pone.0240147 (PMC7943005; doi:10.1371/journal.pone.0240147)
Supplement: S2 Appendix — (PDF) [file pone.0240147.s013.pdf]

S2 Appendix. Akaike's Information Criterion.

For model comparison, we computed Akaike's Information Criterion (*AIC*) [1]. *AIC* allows us to compare performances of models that differ in architecture (e.g., rectification vs. square wave) and in the number of parameters for each model. Specifically, we computed *AIC* for the least square model fitting with the following formula:

$$AIC = n\ln(\sigma^2) + 2K \quad (1)$$

, where n denotes the number of observations, σ^2 denotes the variance of the error term across the observations (in our case, n=156 for S1 and n=101 for S2, see the main text) considered for the model fitting, and K denotes the number of estimable parameters in a model, including the variance of the error term. The error term is the difference that we minimised by optimisation. Then, we computed the σ as the variance of the minimised difference across channels, assuming that the error term is normally distributed with mean = 0.

References

1. Burnham KP, Anderson DR. Multimodel Inference: Understanding AIC and BIC in Model Selection. Sociological Methods & Research. 2004;33(2):261-304. doi: 10.1177/0049124104268644.
