# Supplementary material for: Steady state evoked potential (SSEP) responses in the primary and secondary somatosensory cortices of anesthetized cats: Nonlinearity characterized by harmonic and intermodulation frequencies
Source: PLoS One. 2021 Mar 9;16(3):e0240147. doi: 10.1371/journal.pone.0240147 (PMC7943005; doi:10.1371/journal.pone.0240147)
Supplement: Attachment — Submitted filename: Response to Reviewers.pdf [file pone.0240147.s014.pdf]

MONASH University

Naotsugu Tsuchiya, PhD
Professor
School of Psychological Sciences
Faculty of Medicine, Nursing and Health Sciences

9 January 2021

Dear Dr. Thomas Wennekers,

We sincerely appreciate your time for evaluating our manuscript and considering it for publication in PLOS ONE.

We carefully considered and addressed all comments from the reviewers. Specifically:

1. We clarified various aspects of the data and experiments to address reviewers' comments.
2. We addressed all minor comments from the reviewers.
3. We have made the data publicly available online at OSF (DOI: 10.17605/OSF.IO/CQRFJ)

We also added details on (1) methods of sacrifice, (2) the source of the animals, and (3) details of housing and care in the Methods section.

Regarding the Acknowledgements, the information we provided (the MASSIVE HPC facility) is not a funding source. Therefore, we keep the Acknowledgements as it is.

With these revisions, we believe that our revised manuscript has improved significantly and addressed all the comments. Please also see our point-by-point responses to the reviewers' comments attached below. We believe that the manuscript is now clear and suitable for publication in PLOS ONE.

Regards,

Naotsugu Tsuchiya (on behalf of the coauthors)

Building 220, 770 Blackburn Rd
Monash University, Melbourne, VIC 3800, Australia
Telephone +61 3 9905 4564 Facsimile +61 3 9905 3948
Email naotsugu.tsuchiya@monash.edu

Reviewer #1:

We would like to thank you for all the constructive comments. Below, we provide your original comments in italics and our point-by-point responses in normal font, and our emphasis in **bold** font. Throughout, line numbers refer to line numbers in the revised manuscript.

> *Review of Kawashima et al., "Steady state evoked potential responses in the primary and secondary somatosensory cortices of anaesthetized cats", submitted to PLoS ONE.*
>
> *Summary*
> *This study reports local field potential recordings from cat somatosensory cortex, during vibrotactile stimulation of the paw. This is a very interesting topic, using a technique that is widespread in studying other senses, but which has not yet been fully exploited for somatosensation. The data appear to be of good quality, though I have some queries about the choice of frequencies, and also suggestions about the way the results are presented. There are also some minor comments listed below that should not be difficult to fix or clarify.*

We would like to thank you for taking the time to go through our manuscript. Our point-by-point responses to your comments are as follows:

> *Specific points*
>
> *1. This is an excellent data set. But I feel that it could be presented with much greater clarity. For example,* ***I did not understand the purpose of plotting the % of significant channels (Figure 2)****. As an alternative,* ***why not plot the signal strength (either SNR, power or VELogP, I don't have a particular preference) as a function of the stimulus amplitude?*** *That would allow you to show modulation-response functions. For example, you could plot the amplitude of the 23Hz stimulus on the x-axis and the response at 23Hz on the y-axis, with different functions for different amplitudes of 200Hz stimulation. Then, if the 200Hz component affects the 23Hz response, you will see a 'gain control' type effect. This is the standard way of looking at such data for other senses such as vision (see e.g. Busse et al,. 2009, Neuron; Carandini & Heeger, 2012, Nat Rev Neurosci; Tsai et al., 2012, J Neurosci), and would allow for more straightforward comparisons across studies, as well as being generally clearer.*

Thank you for the suggestion. We now implemented the format that you suggested. We grouped sessions which had the same number of stimulus conditions, and plotted log*SNR* at 23 Hz and 200Hz as functions of stimulus amplitudes for each group. Unfortunately, these figures (to be added as Supplementary Figures 11-13) are not as clear as the examples from the figures of the papers that you recommended. We believe this is due to two reasons. First, as we explained in Table 1, each experimental session varied in stimulus location, amplitudes, duration and # of trials. Possibly due to this variability, second, many recording sites were not responsive to the stimuli, which you can see from Figure 3-5 in the main text, thus including them will dilute the effect substantially. To clarify the response pattern, we could have chosen some arbitrary threshold based on the response amplitude etc, which require adjustment for each recording session (Table 1). Upon considering the clarity and statistical rigour, we concluded that our original Figure 2 as a more valid

summary of the responses of all channels in all sessions. Thus, we modified Fig 2 caption and added your suggested figures as Sup Figs 1-3.

Line 381 - ADD:
We also tried to see a ‘gain control’ type effect by showing logSNR as functions of stimulus amplitudes (Sup Figs 1-3). Unfortunately, the results were not clear possibly due to 1) variations in experimental conditions (Table 1) and 2) spatial specificity in the neural response (See Figs 3-5, below).

Line 1057 (Sup Figs 1-3) - ADD:

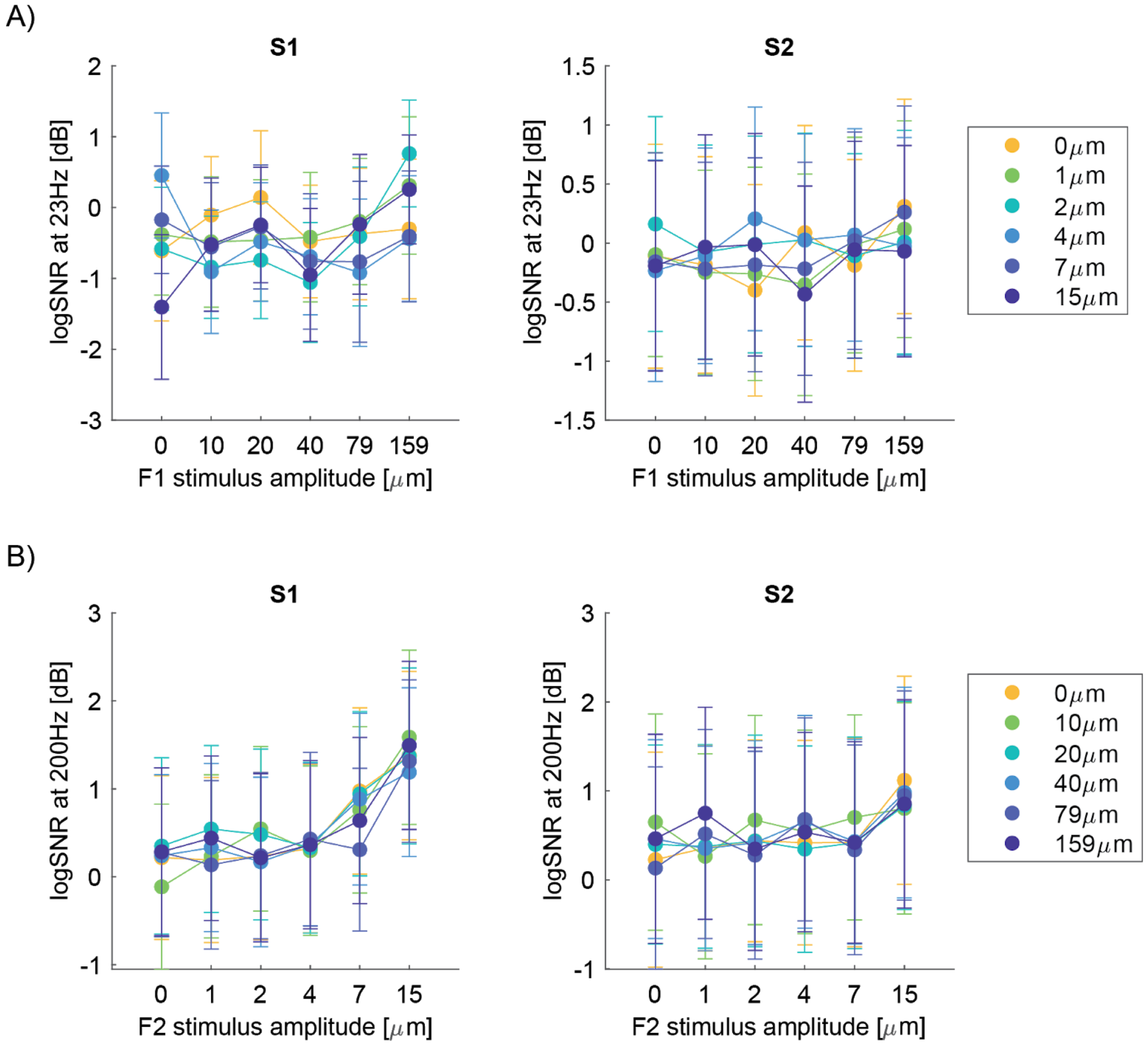

Sup Fig 1. log*SNR* as functions of stimulus amplitudes (# of conditions = 6).
(A) log*SNR* at *f1*=23Hz as functions of *F1* stimulus amplitudes for S1 and S2. We computed the mean and standard error across trials per bipolar channel in Session 1-1 and 1-2, which had 6 amplitude conditions for each *F1* and *F2* vibration stimulus. The point in the graph is the mean across the channels and the error bar is the mean of the standard errors across the channels. Colour encodes *F2* stimulus amplitudes. (B) log*SNR* at *f2*=200Hz as functions of *F2* stimulus amplitudes for S1 and S2. Colour encodes *F1* stimulus amplitudes.

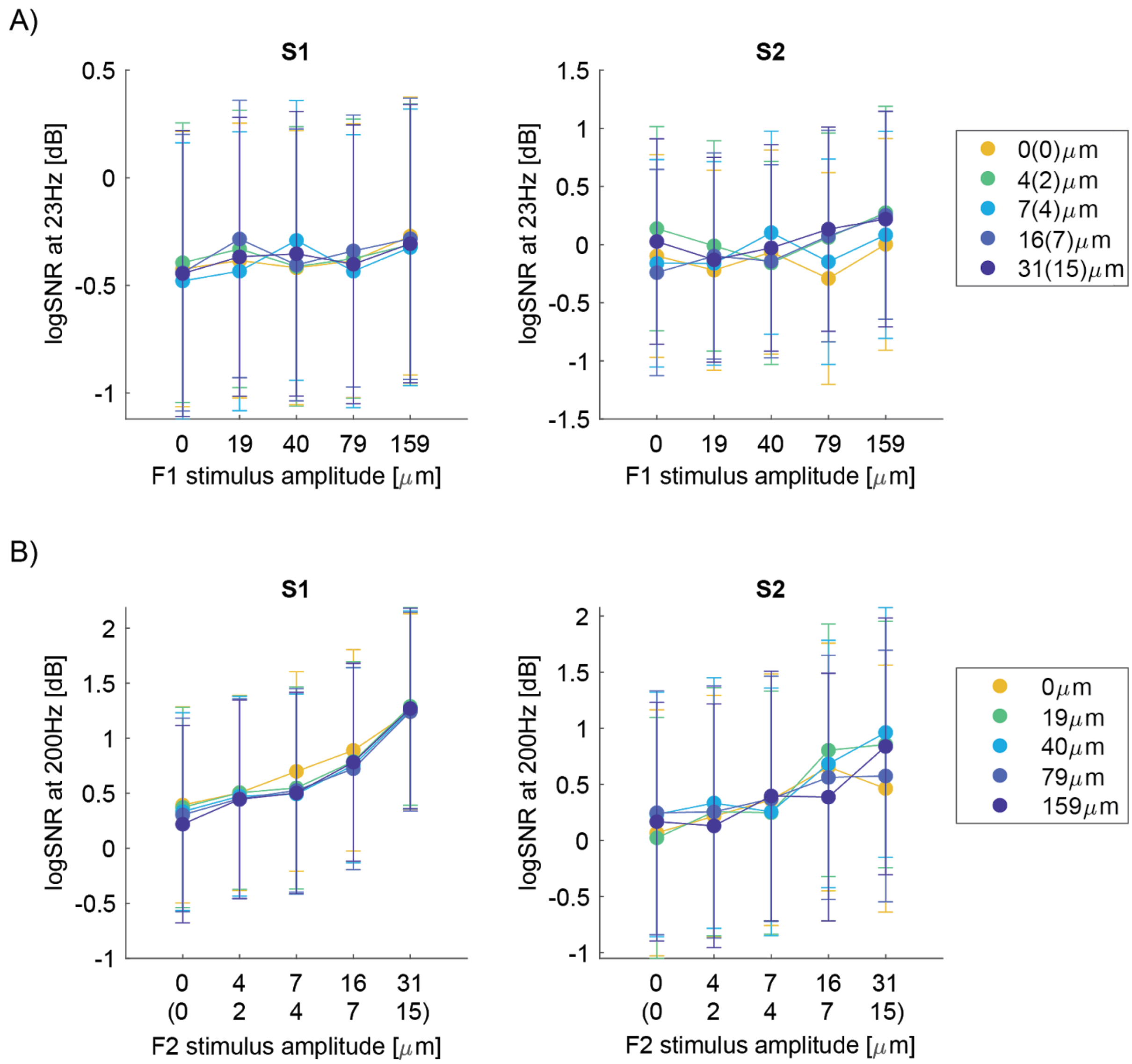

Sup Fig 2. log*SNR* as functions of stimulus amplitudes (# of conditions = 5).
(A) log*SNR* at *f1*=23Hz as functions of *F1* stimulus amplitudes for S1 and S2. The same format as Sup Fig 1. We included Session 1-3, 2-4, 2-5 and 2-6, which had 5 amplitude conditions. Note that Session 1-3 had different *F2* stimulus amplitudes from the other sessions, these values that are shown in brackets. (B) log*SNR* at *f2*=200Hz as functions of *F2* stimulus amplitudes for S1 and S2.

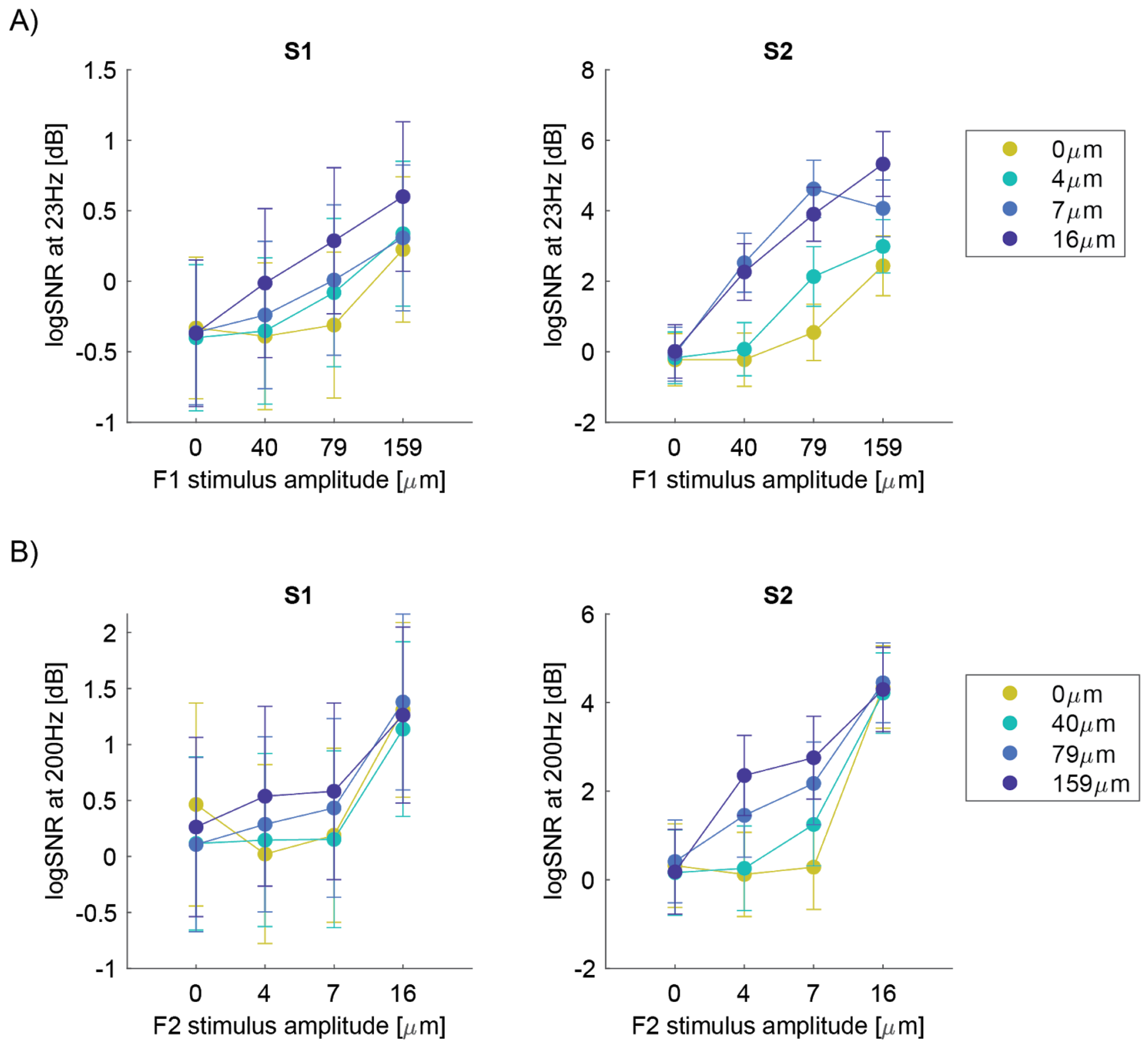

Sup Fig 3. log*SNR* as functions of stimulus amplitudes (# of condition = 4).
(A) log*SNR* at *f1*=23Hz as functions of *F1* stimulus amplitudes for S1 and S2. The same format as Sup Fig 1. We included Session 2-1, 2-2 and 2-3, which had 4 amplitude conditions. (B) log*SNR* at *f2*=200Hz as functions of *F2* stimulus amplitudes for S1 and S2.

> *2. I thought the attempt to model the pattern of intermodulation responses was impressive. However I have not come across the 'square wave' nonlinearity before. Is this something that neurons have plausibly been shown to implement? I would have thought a squaring (or other power law) nonlinearity would be a more obvious choice to investigate, or even full-wave (for comparison with half-wave) rectification.*

We agree that square wave nonlinearity is less plausible in biology. Our original intention was to compare a different type of nonlinearities than half-wave rectification, which were considered in Gordon et al 2019 Neuroimage [15]. We did not include squaring and full-wave rectification because, from Gordon et al 2019, we knew that these operations would effectively "double" the inputs' frequencies and would not generate responses at fundamental frequencies. Thus, these operations can't explain our primary results, that the responses are the combinations of the fundamentals, harmonics, and intermodulations. (Having said that, this does not prove that these two operations are not observed in S1 or

S2 in our paradigm. If they are, they are in combinations with other nonlinearities to explain our results). To make clear these points, we added Sup Fig 10 as follows.

Line 1128 (Sup Fig 10) - ADD:

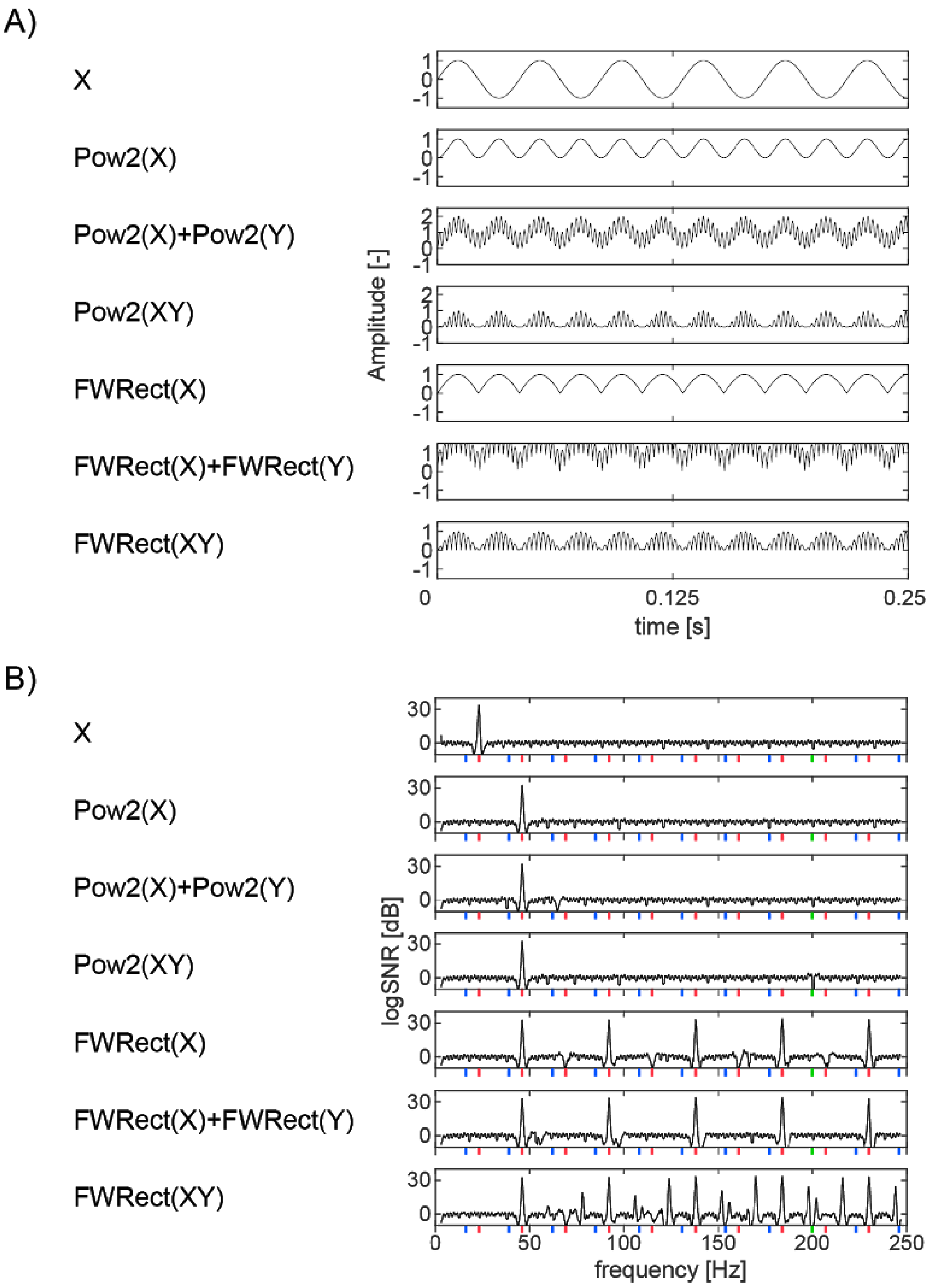

Sup Fig 10. Waveforms of various modelled response types.
(A) Waveforms in the time domain (0 to 0.25s). X and Y are sinusoidal inputs at 23Hz and 200Hz, respectively. Pow2 represents the squaring (i.e. X^2) and FWRect represents the full-wave rectification. Note that, for these operations, multiplication

inside and outside of functions are identical with each other i.e. f(X)*f(Y) = f(X*Y). (B) Spectra of each waveform in the frequency domain.

In the revised manuscript, we instead considered half squaring defined as follows:

HSq(X) = X^2 (X>0)

= 0 (otherwise)

,where X is an input. This operation is biologically plausible, as suggested to model neuronal responses in the visual cortex of cats (Heeger, 1992, Visual Neuroscience [28]). HSq showed a similar result to rectification operation i.e. adding nonlinear operation improved fitting performance specifically in S2. (See Supplementary Figure 5 for details) Compared to rectification, HSq was slightly worse in terms of AIC (See our new Fig 12).

Note that the number of models we considered increased by 2 because the square wave class had 2 models and the half squaring class had 4 models. This changed Bonferroni correction and accordingly changed corrected *p*-values.

Reflecting the above changes, we modified Results, Discussion and Supplementary materials as follows:

Line 548 - MODIFY:
Given the demonstrated biological plausibility [15, 26-28], here we focused on two types of nonlinear processing, that is, rectification and half squaring. For a sinusoidal input *X*=sin(2π*f*t*), where *t* is time and *f* is input frequency, we modelled rectification nonlinearity as,

Rect(X) = X (if X>0)
0 (otherwise)

, and half squaring nonlinearity as,

HSq(X) = X^2 (if X>0)
0 (otherwise)

Line 565 - MODIFY:
We considered two classes of models; one based on combinations of *Rect* and the other based on *HSq*.

Line 585 - MODIFY:
For a given channel, we thus have 8 sets (4 for rectification and 4 for half squaring class) of the best fit coefficients and the minimum difference between model and actual log*SNR*.

Line 603 - ADD:
HSq nonlinearity showed a different pattern of fitting from *Rect* nonlinearity (See Sup Fig7). Specifically, in the case of *HSq* nonlinearity, adding a type of multiplication (i.e. *HSq*(XY)) did not change fitting performance at all.

Line 635 - ADD:
We had a similar observation from the half squaring class except the fact that including *HSq*(*XY*) did not change fitting performance at all. (See Sup Fig 9.)

Line 641 - MODIFY:
Fig 12 shows *AIC* for 8 models, demonstrating the fitting performance is best for the full rectification model for both S1 and S2 when taking into account the number of model parameters. Since adding *HSq*(*XY*) did not change the fitting performance, the models without *HSq*(*XY*) were better than the models with *HSq*(*XY*) when we corrected the bias.

Line 654 - MODIFY:
Although adding an integrative nonlinear components *HSq*(*X*)*HSq*(*Y*) still showed a statistically significant difference between S1 and S2 by Kolmogorov-Smirnov (the right panel in Sup Fig 9), it attenuated the difference between S1 and S2 compared to the simplest model *HSq*(*X*)+*HSq*(*Y*) (the left panel in Sup Fig 9).

Line 766 - ADD:
Although squaring (X^2) and full-wave rectification (abs(*X*)) are also biologically plausible, these operations do not generate responses at fundamental frequencies as they “double” inputs’ frequencies (Sup Fig 10). While the combination of these and other operations (rectification or half squaring See Fig 10) can explain our results, we will not pursue them to restrict the possible model spaces. Thus, we did not consider these operations for modeling analysis.Overall, the quality of model fitting was similar between the rectification class and the half squaring class (Figs 11 and 12 and Sup Figs 7 and 9).

Line 572 (Fig 9) - MODIFY:

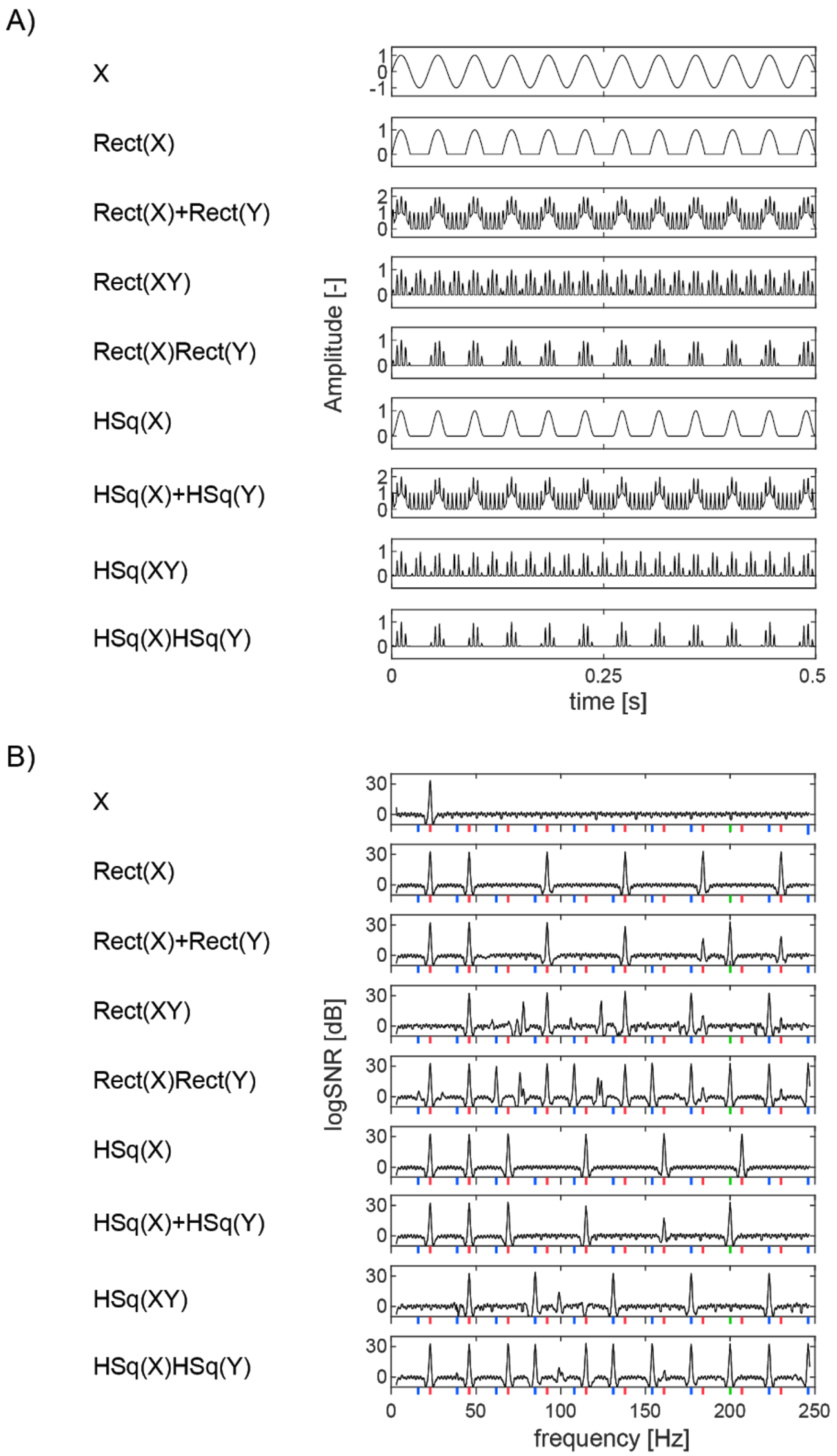

Fig 9. Waveforms of modeled processes.
(A) Waveforms in the time domain (0 to 0.5s). *X* and *Y* are sinusoidal inputs at 23 and 200Hz, respectively. For the definitions of *Rect* and *HSq*, see the main text. (B) Spectra of each waveform in the frequency domain.

Line 577 (Table 2) - ADD:

Table 2. Models considered in analysis

**Model ID**	**Model**
Rect-1	*a*Rect(X)+b*Rect(Y)*
Rect-2	*a*Rect(X)+b*Rect(Y)+c*Rect(XY)*
Rect-3	*a*Rect(X)+b*Rect(Y)+c*Rect(X)Rect(Y)*
Rect-4	*a*Rect(X)+b*Rect(Y)+c*Rect(XY)+d*Rect(XY)*
HSq-1	*a*HSq(X)+b*HSq(Y)*
HSq-2	*a*HSq(X)+b*HSq(Y)+c*HSq (XY)*
HSq-3	*a*HSq(X)+b*HSq(Y)+c* Hsq (XY)*
HSq-4	*a*HSq(X)+b*HSq(Y)+c*HSq (XY)+d*Hsq (XY)*

This table shows all models we considered for our analysis. *a,b,c* and *d* are coefficients to be optimized.

Line 671 (Fig 12) - MODIFY:

A)

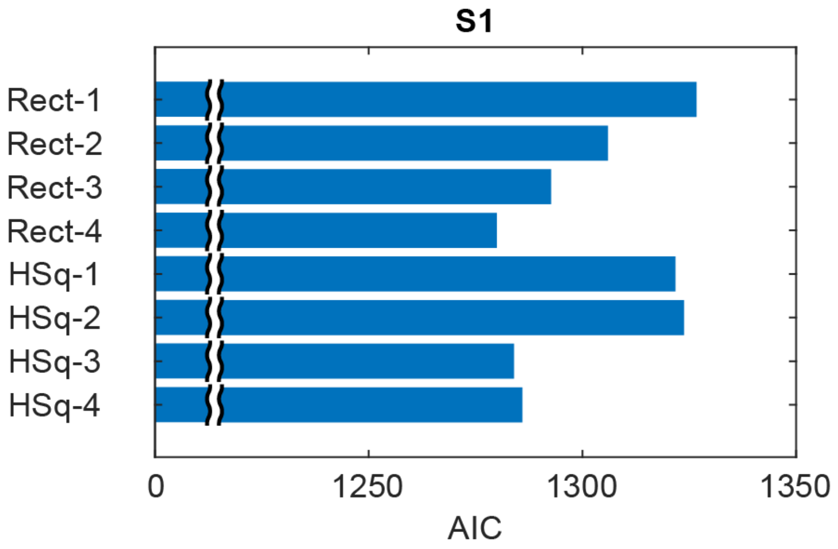

B)

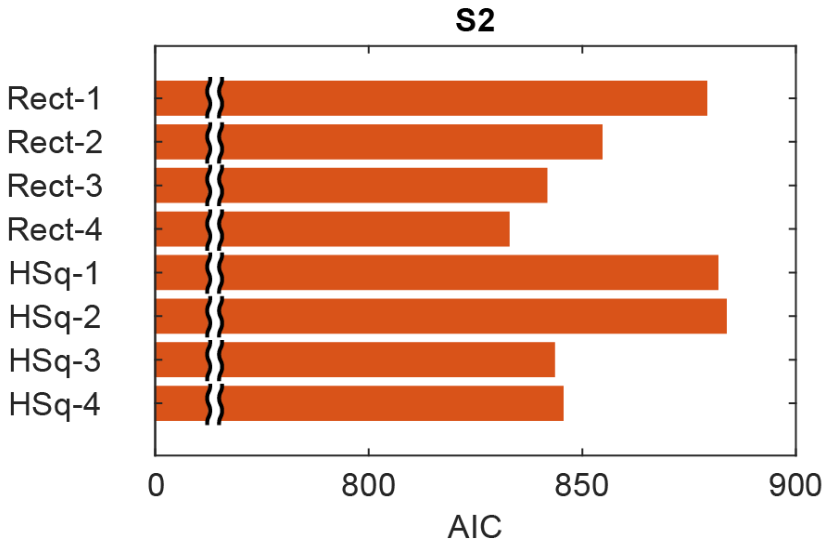

Fig 12. Akaike's Information Criterion (AIC) for 8 models for S1 and S2. The best model is the full rectification model for both S1 and S2.

Line 1097 (Sup Fig 7) - MODIFY:

A)

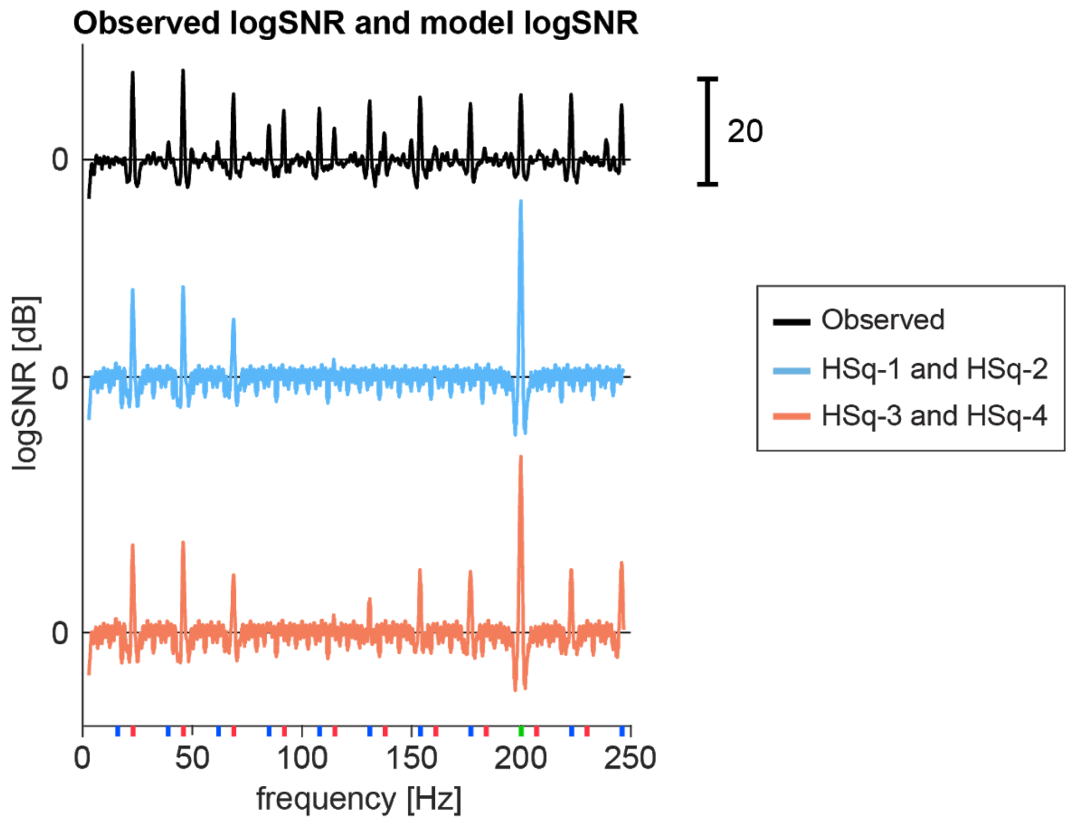

B)

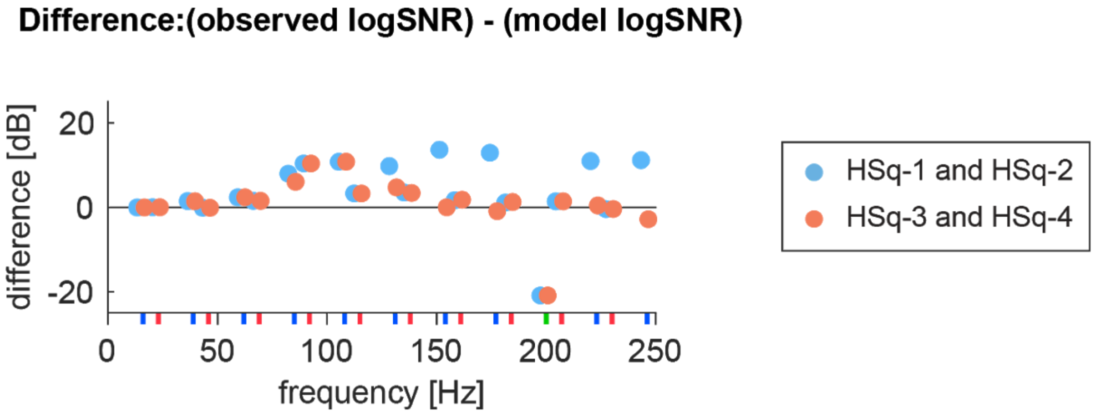

Sup Fig 7. Exemplar channel's observed and modelled responses for half squaring models (HSq).
(A) Bipolar channel 43 in S1 (Session 2-2)'s log*SNR* (black) is compared to the optimally fitted models from four half squaring models. Adding *HSq*(*XY*) to two models (HSq-1 and HSq-3 in Figure 12) did not change the results and we showed four models in two colors: red for HSq-1 & HSq-2 and blue for HSq-3 & HSq-4. The best parameters for each model are the following. HSq-1 (red): -1.0*HSq*(*X*)+64*HSq*(*Y*), HSq-2 (red): -0.54*HSq*(*X*)+ 34*HSq*(*Y*) -1.4*10^{308}*HSq*(*XY*), HSq-3 (blue): -0.94*HSq*(*X*)+38*HSq*(*Y*)+1.4*HSq*(*X*)*HSq*(*Y*) and HSq-4 (blue): -0.85*HSq*(*X*)+35*HSq*(*Y*)-1.4*10^{308}*HSq*(*XY*)+1.3*HSq*(*X*)*HSq*(*Y*). The respective sums

of logSNR differences from the observed logSNR across the frequencies of interest are 125 and 74. (B) Difference between the observed and the best model at the frequencies of interest. Color scheme is the same as in (A).

Line 1119 (Sup Fig 9) - MODIFY:

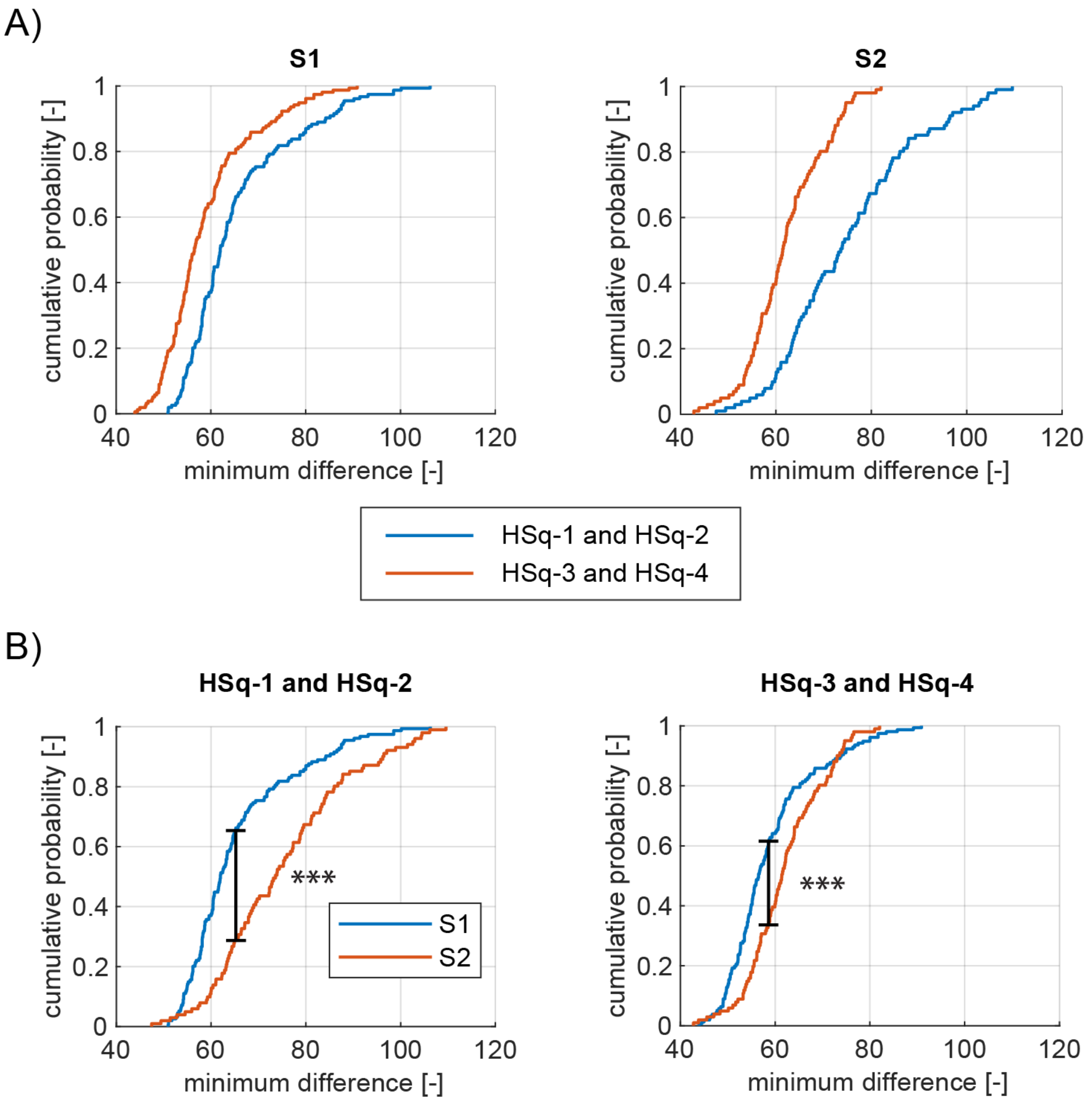

Sup Fig 9. Comparison of fitting performances across half squaring models.
(A) Comparison of performance across four half squaring models based on the cumulative probability distributions of the minimum difference for S1 and S2 separately. Adding *HSq*(*XY*) did not change the results and we showed four models in two colors: red for HSq-1 and HSq-2 and blue for HSq-3 and HSq-4. (B) Comparison of each model's performance between S1 and S2 based on the cumulative probability distributions of the minimum difference. We showed the two models giving the same distribution curves as one subplot. *** indicates $p<0.001$ according to Kolmogorov-Smirnov tests.

Line 1128 (Sup Fig 10) - ADD:
We added the same figure as shown in above.

3. I was surprised that the stimulation frequencies were so different from each other, and that 200Hz was chosen. Q1) Would the authors expect that the same neurons driven by 23Hz stimulation could also respond to 200Hz stimulation, or would these be likely to be distinct populations? Q2) Would you expect very different results if you used more similar frequencies that would be more likely to fall in a single neuron's frequency response, such as 23Hz and 27Hz, or 200Hz and 187Hz? Q3) Why was 200Hz chosen in the first place, given that cortical neurons typically do not respond this fast (as the authors point out in the Discussion)?

The data of this paper originally came from a series of experiments that set out to investigate how the early somatosensory system organizes and integrates tactile information from distinct sensory endings encoding vibrations at ~23 and ~200 Hz respectively at the finger tip (Carter et al., 2014, PLoS One [16]). In this paper, joined by the expertise of the first and last authors, we continue to examine the cortical processing behind the ubiquitous sensation of touch and tactile discrimination from a computational perspective. Indeed, it was very striking to us as well to find strong intermodulation phenomena (Gordon et al., 2019, Neuroimage [15]) in the cortical processing of somatosensory information with this level of separation (23Hz vs 200Hz) in stimulation frequencies, which, to our knowledge, has not been reported before.

As to your specific questions:

Q1) Do the same neurons driven by 23Hz stimulation could also respond to 200Hz stimulation? Or distinct populations?

According to our previous study (Carter et al., 2014, PLoS One [16]) which examined single neuron responses, most neurons were sensitive only to either 23 Hz or 200 Hz. A small population of neurons' responses are nonlinearly modified by the presence of the other frequency. Evidence from Saal, H. P., Harvey, M. A., & Bensmaia, S. J. (2015). Rate and timing of cortical responses driven by separate sensory channels. *Elife*, *4*, e10450, suggests that "the vast majority of cortical neurons [in S1] receive (indirect) input from both RA and PC afferents".

Q2) Different results if different frequencies were used??

Most likely, yes, but we have no data to support such a statement. However, we expect that a pair of more similar frequencies might induce intermodulation at the earlier levels of processing, possibly before S1 or S2.

Q3) Why was 200Hz chosen in the first place?

200 Hz was utilized to selectively stimulate very fast-adapting, so-called, Pacinian (PC or RAII), sensory endings in the cat's glabrous skin (paw pad), as opposed to 23 Hz for targeting rapidly-adapting (RAI) sensory endings. Having said that, we did not expect that 200Hz stimulation would induce 200Hz responses as seen in the LFP. In fact with our single neuron study (Carter et al., 2014, PLoS One [16]), we never found any neurons that fired at 200Hz as we explained in Discussion.

We clarified these three points in Methods and Discussion as follows:

Line 165 - ADD:
We chose 23Hz and 200Hz to stimulate rapidly-adapting sensory endings (RAI) and very fast-adapting, so-called Pacinian (PC or RAII) respectively.

Line 793 - ADD:
In this study, we investigated across-submodality interactions by using a pair of stimulus vibration frequencies (23Hz and 200Hz) which stimulates two different sensory endings: rapidly-adapting sensory endings (RAI) and very fast-adapting (PC or RAII). It is also possible to investigate within-submodality interaction by using a pair of frequencies which stimulates the same sensory endings (e.g. stimulate RAI by 25Hz and 40Hz), which might result in intermodulation even at the level before the cortical processing, resulting in different power spectrum patterns. If close tagging frequencies were used, some nonlinearities may originate at subcortical levels. Indeed, some harmonic responses were reported at the median nerve in humans while presenting vibration stimulus [44]. Homologous findings can be found for the intermodulations as well.

> *4. Typically, frequencies beyond the pass-band of a filter can produce lower frequency aliasing artifacts. Might the broadband response be explained by aliasing artifacts from a population of neurons that all have slightly different temporal properties, being driven by a high frequency beyond their usual range (e.g. 200Hz)?*

We share the reviewer's feeling of surprise in finding the high-gamma broadband responses in S2. We ourselves did not initially understand this response, but it was not caused by any types of artifacts that the reviewer suggests because we did not perform any band-pass filtering. Furthermore, we did not observe HGP responses in S1 even though we performed the same data analysis for the recording in S1 and S2.

To confirm the observed HGP was not any type of artifacts, for a wider range of frequencies, we plot *VELogP* response (deviation of logPower responses from no stimulus conditions). Sup Fig 11 shows mean *VELogP* from the top 10% channels showing higher HGP responses in S2.

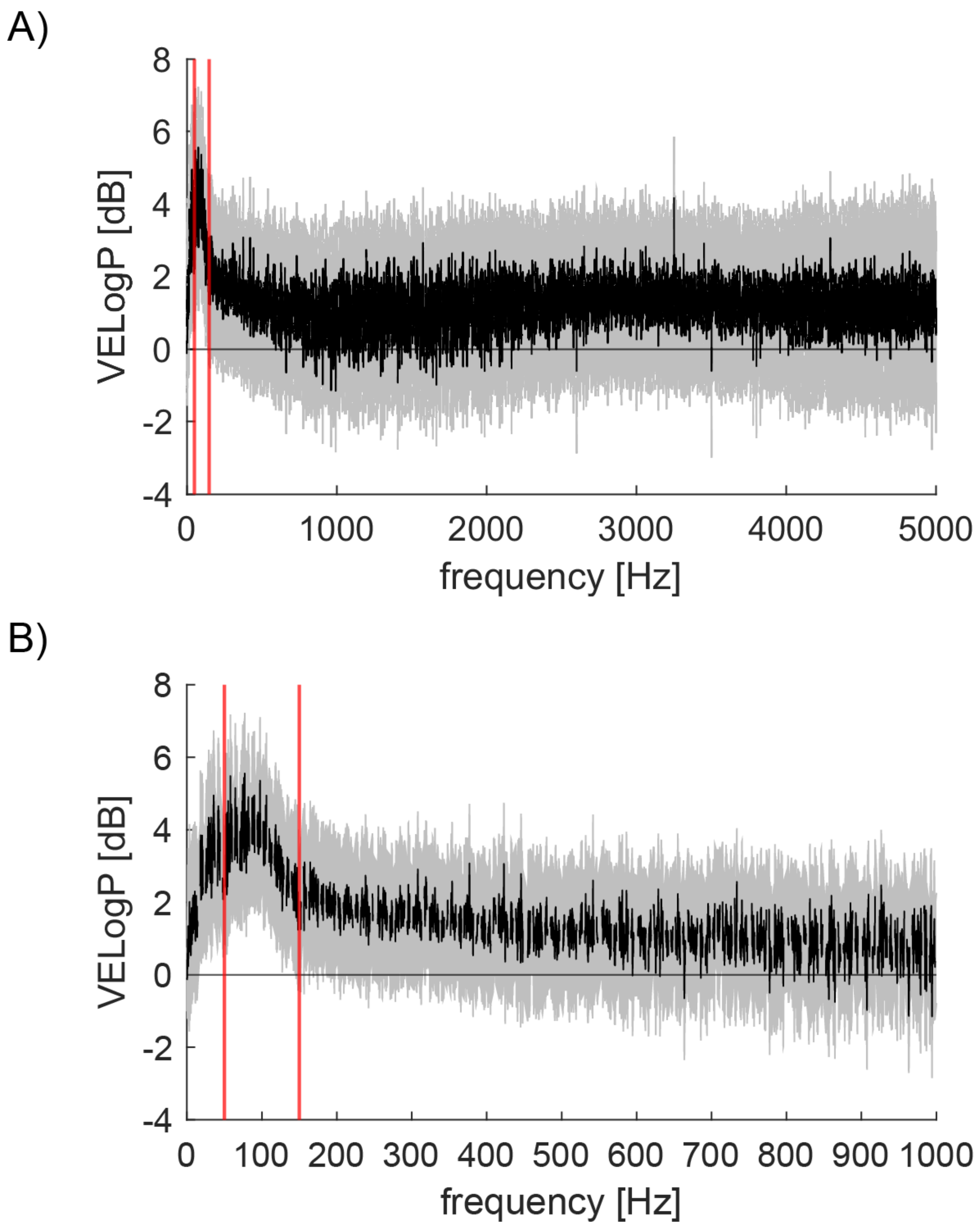

Sup Fig 11. *VELogP* from top 10% channels in S2 across A) 0-5000Hz and B) 0-1000Hz). The top 10% channels showing higher HGP responses in S2. We first took mean and standard error across trials for each channel and then took mean across channels. Red vertical lines show 50Hz and 150Hz. For a display purpose, we removed responses at fundamentals, harmonics and some intermodulation frequencies.

As you see, a specific increase from 50-150Hz is genuine and not present in any other multiple of 50-150Hz beyond 250Hz (even up to 5,000 Hz). Note that the *VELogP* format reveals slightly different peaks than 50-150Hz (more like 25-125Hz), but this is not essential

for our claim. To remind the reviewer, these ‘non-tagged’ responses are very surprising from the view point of the SSEP paradigm, which assumes that there should be no such responses.

One explanation we came up with is that this is a reflection “high gamma” band response (Canolty et al., 2006, Science [19]; Tsuchiya et al., 2008 PLoS One [49]) (not the non-specific, broadband responses mentioned in Miller, K. J., Zanos, S., Fetz, E. E., den Nijs, M., & Ojemann, J. G. (2009). Decoupling the Cortical Power Spectrum Reveals Real-Time Representation of Individual Finger Movements in Humans. *Journal of Neuroscience*, 29(10), 3132-3137).

We added the above Sup Fig 11 to Supplementary material and clarified your point in Discussion as follows:

Line 1139 (Sup Fig 11) - ADD:
We added the figure in the above.

LIne 830 - MODIFY:
One might consider the observed broadband (50-150Hz) HGP as aliasing artifacts that band-pass filtering produces in its lower frequency range. We believe the observed HGP is unlikely to be due to the artifacts for four reasons. First, we did not perform any band-pass filtering to the recording in S1 and S2. Second, HGP were observed only in S2 not in S1 even though we performed the same analysis for S1 and S2 recordings. Third, we did not observe strong responses at any other multiples of the HGP range even up to 5000Hz (Sup Fig 11). Fourth, such broadband HGP around 50-150Hz has been reported in some studies that analyzed ECoG [47-49] and LFPs [50].

Having said that, the HGP responses documented in these studies tend to be short-lived and to disappear soon after the stimulus onset. Thus, the evoked HGP may not be relevant for log*SNR* computation if only the sustained components are used. However, our S2 HGP were in fact sustained and at the comparable magnitude with those at the harmonic and intermodulation frequencies (Fig 8).

> *5. The data availability statement says that the data will be made available after acceptance of the manuscript. I believe that assessing data quality and availability is an important part of peer review, and I think that the data should at least be made available to the reviewers upon resubmission (OSF permits this with ‘read only’ links).*

We apologize for this oversight and thank you for letting us know that OSF has such a function. We did not know it at the submission. We now made the data fully available at this point. (DOI: 10.17605/OSF.IO/CQRFJ)

> *6. Line 149 (and elsewhere), I didn’t understand the need for inverted commas around “deviation”.*

We agree with you. We removed the quotation marks everywhere applicable as follows:

Line 156 - MODIFY:

Shade represents the standard deviation across 15 trials, indicating extremely robust and clean SSEP.

Line 158 - MODIFY.
Again, shading represents standard deviation.

> *7. Conversions to power and logSNR are described clearly. But in the figures, logSNR is expressed in dB. Conventionally this involves multiplying the raw log values by some factor (usually 10 or 20), but I couldn't see this specified anywhere.*

Thank you for pointing this out. In our conversion method, we multiplied raw log values by 10. We clarified it in Methods as follows:

Line 161 - ADD:
Note that raw log values are multiplied by 10 and values are shown in [dB].

Line 279 - ADD:
Note that raw log values are multiplied by 10 and values introduced here are shown in [dB].

> *8. The rationale for the analysis given in Figure 2 was rather unclear. Why should we care about the % of channels that show one or other response, or their interaction? It wasn't obvious to me what this was telling us.*

This point is answered in response to your Specific Point 1; Due to the heterogeneity of the stimulus conditions etc in Table 1 and neural responses demonstrated in Figure 3-5, we believe that % of channels that demonstrate these responses at a fixed statistical threshold is the most logical. In particular, Figure 2 shows that less than 15% of recorded sites show the effects, but when they show the effects they are very clear and strong responses. This is important information for those who are working on less precise neural recording modalities than LFP, such as EEG or fMRI. We clarified it in Results and Discussion as follows:

LIne 340 - MODIFY:
The statistical analyses revealed that responses at the frequencies of interest were not observed across all channels and specific to some channels (Fig 2).

Line 811 - MODIFY:
Compared to such coarse resolution, the spatial resolution of our findings is quite striking. For example, Fig 2 shows that not all channels reflected SSEPs, suggesting at least the equivalent level of spatial resolution is necessary for localizing their sources. (See Figs 3-5 and 7 as well).

> *9. If each trial is 2 seconds long, the spectral resolution should be 1/2 = 0.5Hz, as stated on line 210. But it appears to be much higher than this in Figure 3A. Why?*

The reviewer is correct in terms of the spectral resolution. However, Figure 3A involved SNR computations, which effectively smoothed the spectra as we explain below.

Specifically, we computed log*SNR* at a frequency by subtracting mean logPower at its neighbouring frequencies from the one at the frequency. As the neighbor frequencies, we used a set $F' = \{f \mid f-3<f'<f-1 \text{ or } f+1<f'<f+3\}$. To check the smoothing effect due to the

neighboring frequencies, we compared log*SNR* and vibration evoked logPower (*VELogP*) in Fig Reb 1, which shows responses at 23Hz at bipolar channel 131 in S1 from the maximal stimulus condition (corresponding to the bottom right panel in Fig 3A). As expected, log*SNR* is smoothed compared to *VELogP* that has the spectral resolution of 0.5Hz.

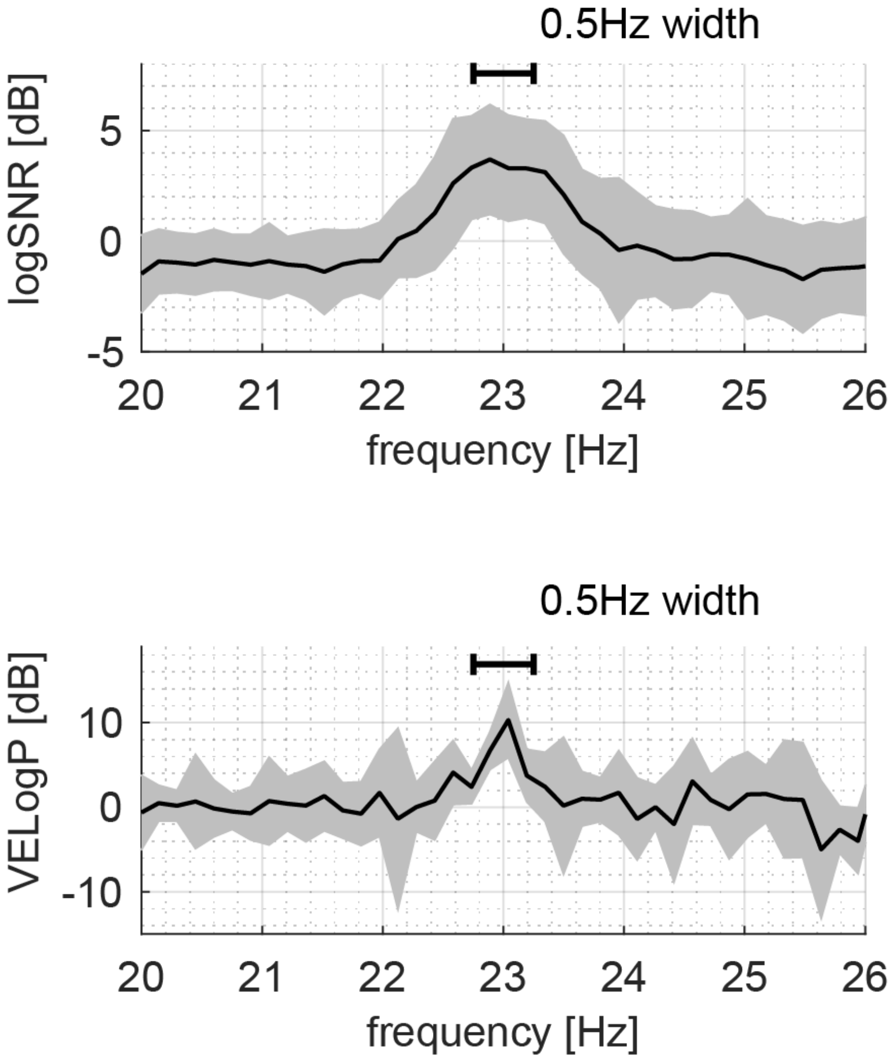

Fig Reb 1. Exemplar log*SNR* and *VELogP* at 23Hz at bipolar channel 131 in S1.
Mean responses across trials in the maximal stimulus condition in Session 2-2. Grey shading represents standard deviation. Log*SNR* is smoothed compared to *VELogP* that has the spectral resolution of 0.5Hz.

We clarified this in Results as follows:

Line 409 - ADD:
Note that, as we considered a set of frequencies $F' = \{f \mid f\text{-}3<f'<f\text{-}1 \text{ or } f+1<f'<f+3\}$ as the neighboring frequencies for the log*SNR* computation, log*SNR* is smoothed and has a lower spectral resolution than the half bandwidth of 0.5Hz.

10. The top left panel of Fig7a (and Supplementary Figs 1-3) shows a completely flat line, but with grey error regions similar to the other panels. Is this a plotting error? It looks fine in Figs 3-5.

No, it is not an error. In Fig 7A and Supplementary Figs 1-3, we show vibration evoked logPower (*VELogP*), which we define as the deviation of logPower from the mean logPower in no vibration condition. This captures how a sensory input (i.e. vibration) makes a difference in logPower from the baseline, no-vibration condition.

Thus, if we take the "mean" of VELogP for the no vibration condition (the top left panel in Fig 7A), it should be 0. However, if we take the variance, it is not 0. We clarified this by adding the following sentences to Methods and Results.

Line 258 - ADD:
This captures how presenting vibration makes a difference in logPower from the no-vibration condition.

Line 491 - ADD:
Note that the top left panel, which corresponds to the no-vibration condition, gives a flat line because *VELogP* is defined as the deviation of logPower from mean logPower of no vibration condition. It still has some variance across trials, shown as standard deviation (grey shading).

11. Very hard to see what's happening clearly in Figure 10a. I'd suggest offsetting each trace vertically by some fixed amount, so that we can see all of them clearly.

Thank you for the suggestion. We replaced the previous figure with a vertically offset version in Results as follows:

Line 608 (Fig 10) - MODIFY:

A)

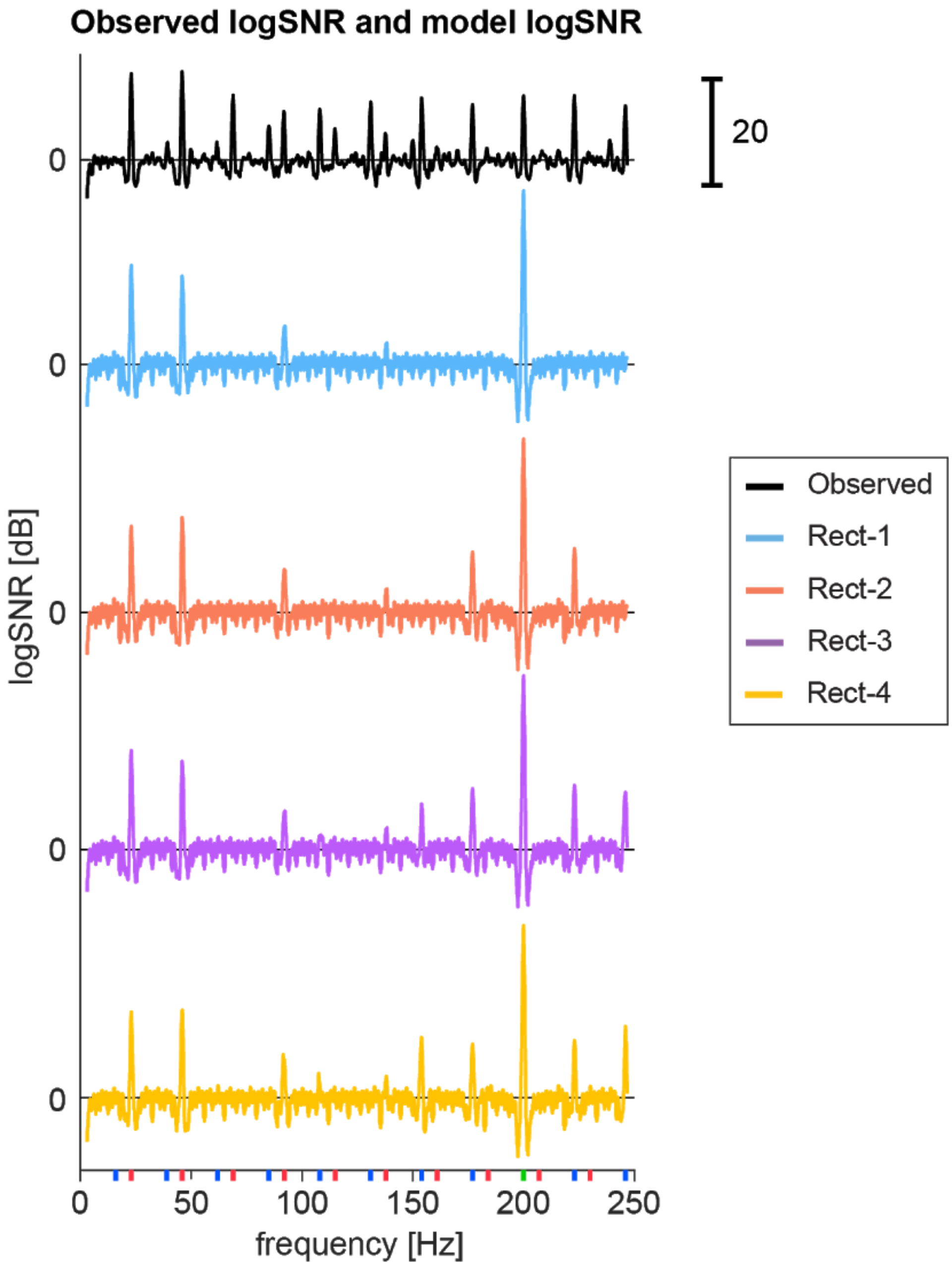

12. I would like to see a clear description of what the cumulative probability curves in Figure 11 (and Supplementary Figure 5 & 6) represent.

We apologise for our lack of explanation.

For Fig 11, as well as Sup Fig 9 (the previous Sup Fig 5), we use the cumulative probability distribution curves to represent that goodness of the fit for each model.

Note that x-axis is the minimum difference between the model prediction and the actual response across bipolar channels. Thus, a channel that has better fit locates closer to 0 in this format. (We can show the same information with histograms. However, histogram

appearance depends on the bin size, thus we opted for the cumulative histograms). By comparing the curves, we tried to see overall differences in fitting performance among models (Fig 11A) and between S1 and S2 (Fig 11B).

In Sup Fig 8 (the previous Sup Fig 6), we used the cumulative probability curves to summarise best fit parameters in rectification models.

Perhaps, the confusion arose because we labelled y-axis as “probability” in the previous manuscript. We relabelled them into “cumulative probability” in the revised manuscript. For the clarification, we modified Results and captions in Supplementary materials as follows:

Line 619- MODIFY:
Next, we summarized the population level statistics of the model fitting. Fig 11 shows the cumulative probability distribution curves of the minimum difference across bipolar channels for each rectification model. The better a model is able to fit responses overall across channels, the quicker its cumulative distribution curve starts in the x-axis and reaches the top i.e. probability = 1 in the y-axis. (See Sup Fig 9 for the half squaring class.)

To address the question of whether integrative computation is observed more frequently in S2 than S1, we compared the model fitting results between S1 and S2 (the left and right panel in Fig 11A). For S1 (the left panel), all curves appeared to similarly start in the x-axis and reach the top i.e. all models appeared to be almost equally fitted to responses in S1. For S2 (the right panel), on the other hand, some curves reached the top quicker than the others i.e. some models were able to fit better than the others. Specifically, the simplest *Rect(X)+Rect(Y)* model, depicted by the blue lines, has a more similar curve to the other models for S1 responses than for S2 responses. Adding another integrative nonlinear *Rect(XY)* component (orange lines) nearly saturated our model performance for S1, while it still left room for improvement for S2. Additional improvement was achieved when we considered the *Rect(X)Rect(Y)* component (purple and yellow), which led to an improvement especially in S2.

Line 647 - ADD:
Finally, we confirmed the statistical difference of the cumulative probability distributions between S1 and S2 for each model (Fig 11B).

Line 654 - MODIFY:
Although adding an integrative nonlinear components *HSq*(*X*)*HSq*(*Y*) still showed a statistically significant difference between S1 and S2 by Kolmogorov-Smirnov (the right panel in Sup Fig 9), it attenuated the difference between S1 and S2 compared to the simplest model *HSq*(*X*)+*HSq*(*Y*) (the left panel in Sup Fig 9).

Line 1110 (Sup Fig 8 (the previous Sup Fig 6)) - MODIFY:
This summarizes actual values of each parameter in rectification models. It also shows how adding a nonlinear component had an effect on other parameters. e.g. the top left panel in A shows that the model with *Rect*(*X*) (purple line) and the full model (yellow line) seemed to have similar the parameter a (i.e. coefficient of *Rect*(*X*) overall. On the other hand, the other two models (blue and red) seemed to have different parameters overall.

Reviewer #2:

We would like to thank you for all the constructive comments. Below, we provide your original comments in italics and our point-by-point responses in normal font. Throughout, line numbers refer to line numbers in the revised submission unless otherwise specified.

> *The manuscript by Tsuchiya et al. explores nonlinear nature of mechanoreceptor input transformation in cat's S1 and S2. The basic methodological approach they use is straightforward: if a dynamical system is strictly linear, its output in response to a stable sinusoidal input signal will be a signal of the exactly the same frequency and varying only in its amplitude and phase shift. In the frequency domain, the power spectrum of such output signal will have only a single spike, located at the input frequency. Furthermore, if a linear system is driven by a sum of 2 or more sinusoidal inputs of different frequencies, the output power spectrum will be a sum of power spectra of constituent frequencies. In contrast, if a dynamical system is nonlinear, there will be additional spikes in the power spectrum at various harmonics of the input frequency, at intermodulation frequencies, etc.*
> *Approaching somatosensory system as a dynamical system in this theoretical framework, the authors interrogated it by (1) driving it with peripheral sinusoidal inputs; (2) using LFPs, recorded at various locations in S1 and S2, as the system's output signals; and (3) displaying these output signals in the frequency domain. Peripheral input signals were in a form of sinusoidal vertical skin displacement oscillations applied to the central pad or the pads of D4 or D5 at a combination of 23Hz and 200Hz frequencies, using different amplitude pairings (ranging from zero to modest strength). Not surprisingly – since we already know that the nervous system is profoundly nonlinear – power spectra of cortical stimulus-evoked LFPs had plenty of spikes at harmonics and intermodulation frequencies. Different recording sites produced a variety of power spectra, some of which might be due to their location in either S1 or S2, or being in the center or margin of the responding cortical region, or particular cortical layer.*
> *The manuscript is clearly written, data analyses and figures are informative and, overall, the paper offers an effective demonstration of the usefulness of this steady-state frequency-domain technique for interrogating somatosensory cortical networks and computational operations they perform on their afferent inputs (which can be used in practical experimental validation of cortical models).*

Thank you very much for your accurate summary and appreciation of our intention!

> *Specific Comments*
>
> *(1) Some of nonlinearities observed in S1 and S2 power spectra might originate at subcortical levels. For example, Kelly et al. (Exp. Brain Res. 109:500-506, 1996) recorded activity of 1st order afferents in human median nerve while delivering flutter stimulation to D2 at 33Hz. Their power spectrum shows a clear 1st harmonic (see their Fig. 1) only about 50% smaller (on log10 scale) than at the driving frequency. This power spectrum is very similar to what you get after rectifying a cosine function.*

Thank you for the reference. We agree that some nonlinearities might originate at subcortical levels. We stated this possibility in Discussion as follows:

Line 799 - ADD :

If close tagging frequencies were used, some nonlinearities may originate at subcortical levels. Indeed, some harmonic responses were reported at the median nerve in humans while presenting vibration stimulus [44]. Homologous findings can be found for the intermodulations as well.

> *(2) Lines 541-542, also Fig. 9: The text states: “As is reviewed by Gordon et al. 2019, these nonlinear operations, Rect(X) and Sq(X), alone do not generate responses at harmonic nor intermodulation frequencies (Fig 9)”. Actually, Rect(X) does generate a substantial response at the 1st harmonic. Gordon et al. (2019) example in fact shows that harmonic clearly. Fig. 9B for Rect(X) in this manuscript shows spikes of equal magnitudes at all odd harmonics, which is not right.*

Thank you for pointing out this typo. You are correct that *Rect(X)* does generate responses at harmonic frequencies. We meant the operation did not generate responses at intermodulation frequencies.

With respect to the discrepancy between our Fig 9B and Gordon’s figure, the difference comes to a minor difference in the modelled responses. Our Fig 9 used a pure sinusoidal input, whereas Gordon et al. 2019 added substantial noise to a sinusoidal input. This subtle difference resulted in the difference that you pointed out. To clarify, we added Sup Fig 10 which shows adding noise to the input of *Rect(X)* resulted in disappearance of these responses, and accordingly we modified Results as follows:

Line 562 - MODIFY:
As shown in Fig 9, these nonlinear operations alone do not generate responses at intermodulation frequencies while they do so when combined with further operations, such as multiplication.

Line 1128 (Sup Fig 10) - ADD:

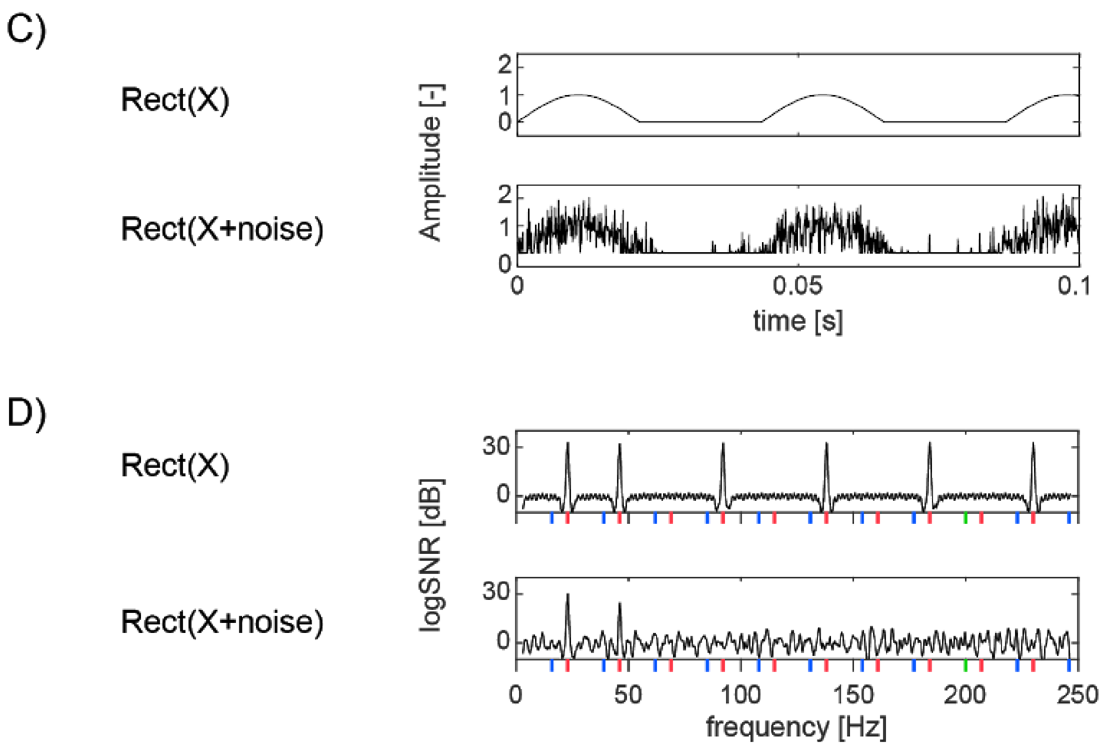

Sup Fig 10. Waveforms of various modelled response types.
(C) Noise can suppress responses at harmonic frequencies in Rect(X).
Waveforms in the time domain (0 to 0.1s). Noise is given by normal distribution *N*(0, 0.52) (as in the simulation reported in Gordon et al 2019). (D) Spectra of each waveform in the frequency domain. Adding noise to the input suppresses responses at some harmonic frequencies.

> *(3) The two stimulus frequencies used in this study (23Hz flutter and 200Hz vibration) preferentially engage different afferent submodalities (RA1 and Pacinian), which have different projections to somatosensory cortex. Power spectrum patterns might change significantly, compared to the ones presented in this paper, if the two stimulus frequencies are confined to the same submodality (e.g., 25Hz and 40Hz). Within-submodality interactions in S1 and S2 cortical columns might be different from across-submodality interactions, and this frequency-domain technique might be able to demonstrate this difference very nicely.*

We totally agree with this. We added this component in Discussion as follows:

Line 793 - ADD:
In this study, we investigated across-submodality interactions by using a pair of stimulus vibration frequencies (23Hz and 200Hz) which stimulates two different sensory endings: rapidly-adapting sensory endings (RAI) and very fast-adapting (PC or RAII). It is also possible to investigate within-submodality interaction by using a pair of frequencies which stimulates the same sensory endings (e.g. stimulate RAI by 25Hz and 40Hz), which might result in intermodulation even at the level before the cortical processing, resulting in different power spectrum patterns. If close tagging frequencies were used, some nonlinearities may originate at subcortical levels. Indeed, some harmonic responses were reported at the

median nerve in humans while presenting vibration stimulus [44]. Homologous findings can be found for the intermodulations as well.

(4) According to Abstract, the cats were studied under ketamine-xylazine general anesthesia, but on page 4 (line 127) it is stated that anesthesia was maintained with alfaxalone. Which one was it?

Sorry for the confusion. The anesthesia was induced with Ket/Xyl (IM). Once the animal was in stereotaxis, anesthesia was maintained with alfaxalone IV for the duration of the experiment. We modified Abstract as follows:

Line 48 - MODIFY:
Here, we analyzed the steady state evoked local field potentials (LFPs) recorded from the primary (S1) and secondary (S2) somatosensory cortex of anesthetized cats (maintained with alfaxalone) while we presented slow (*F1*=23Hz) and fast (*F2*=200Hz) somatosensory vibration to the contralateral paw pads and digits.

(5) According to Whitsel and colleagues (Tommerdahl et al. J. Neurophysiol. 82:16-33, 1999; Tommerdahl et al., Somatosens. Mot. Res. 22:151-169, 2005), Pacinian projections to S1 have prominent suppressive effects on RA1-evoked activation there. No such suppression of the 23Hz stimulus by the 200Hz stimulus is evident in this paper. Some discussion of this disagreement would be desirable. The only significant difference that I can see between the experimental conditions is the anesthetic used: Whitsel et al. used 50/50 nitrous oxide in oxygen (and neuromuscular block) for a very light level of anesthesia, whereas this study used either ketamine or alfaxalone. If it was ketamine, then it might be responsible, since ketamine greatly reduces stimulus-evoked dynamics in S1.

The result here is consistent with our previous publication (Carter et al., 2014, PLoS One [16]), where we found little evidence of the same reported suppressive effect, rather a small degree of nonlinear facilitative effect. We believe the difference in our result is from the methodology used to acquire the physiological signal. We used Utah arrays and analyzed the multi-unit activity (Carter et al., 2014, PLoS One [16]) and LFPs in this paper, whereas Tommerdahl et al used optical imaging of intrinsic signals (OIS). Both approaches are not perfect. OIS interprets neural activity indirectly from the slowly changing oxygenation level of the cortex, and multi-unit/LFP activity integrates both excitatory and inhibitory neural firing. Also, the stimulation duration in our experiments are short (~4s) compared to Tommerdahl et al (~10s). We believe that our electrophysiological approach captures the neural response more accurately to dual-frequency stimulation at the cellular level within a short time from the onset of the dual-frequency stimulus. We revised Discussion as follows:

Line 737 - ADD:
It should be noted that Pacinian afferents to S1 have been reported to suppress RA1-evoked activation [36, 37], but we did not observe such effects clearly (See Sup Figs 1-3), which is consistent with our previous study on multi-unit activity [16]. The difference might be due to the difference in data acquisition. While we directly measured electrical activity, Tommerdahl et al indirectly measured activity as change in oxygenation level of the cortex. Also, we used a shorter stimulus duration than those studies and results in our study reflected activities within a shorter time from the onset of stimulus.

(6) Line 299: change “LEP” to “LFP”

Thank you for pointing this out.

(7) Line 587: “30Rect(X)” is probably “30Rect(Y)”

Thank you for pointing this out.

(8) Line 613: change “Smironv” to “Smirnov”

Thank you for pointing this out.

(9) Line 855: reference #20 uses first names of the authors rather than their surnames. So, Gyorgy, Costas, Christof should be changed to Buzsaki, Anastassiow, Koch.

Thank you for pointing this out. We ensured that the similar errors were corrected in the rest of references for the revised manuscript.
